# Hypomagnetic Conditions and Their Biological Action (Review)

**DOI:** 10.3390/biology12121513

**Published:** 2023-12-11

**Authors:** Ruslan M. Sarimov, Dmitriy A. Serov, Sergey V. Gudkov

**Affiliations:** Prokhorov General Physics Institute of the Russian Academy of Sciences, Vavilove St. 38, 119991 Moscow, Russia; rusa@kapella.gpi.ru (R.M.S.); dmitriy_serov_91@mail.ru (D.A.S.)

**Keywords:** hypomagnetic field, magnetic zero, magnetoreception, cell biology, human physiology, near null magnetic field, Helmholtz system

## Abstract

**Simple Summary:**

The Earth’s magnetic field is vital for life to exist. If the field becomes weaker, it’s known as hypomagnetic conditions. Studying the impact of hypomagnetic conditions on living beings is significant for multiple reasons. Firstly, it helps us comprehend the biological consequences and learn more about how the magnetic field interacts with living organisms. Secondly, understanding the impact of hypomagnetic conditions on human health is important for preparing for extended space missions. This report outlines the influence of hypomagnetic conditions on various objects such as animals, humans, plants, bacteria, and individual molecules. It explains the effects at both a cellular and organismal level, and lists and characterizes the most likely mechanisms that account for biological responses to magnetic fields. Over the past century, scientists have gathered extensive data on the impacts of hypomagnetic conditions. We aimed to investigate the effect of experimental methods and type of exposure on the observed effects. Our findings indicate that hypomagnetic conditions primarily affect cellular processes such as gene expression and protein synthesis, as well as the functioning of the nervous system including neuron development and behavioral reactions.

**Abstract:**

The geomagnetic field plays an important role in the existence of life on Earth. The study of the biological effects of (hypomagnetic conditions) HMC is an important task in magnetobiology. The fundamental importance is expanding and clarifying knowledge about the mechanisms of magnetic field interaction with living systems. The applied significance is improving the training of astronauts for long-term space expeditions. This review describes the effects of HMC on animals and plants, manifested at the cellular and organismal levels. General information is given about the probable mechanisms of HMC and geomagnetic field action on living systems. The main experimental approaches are described. We attempted to systematize quantitative data from various studies and identify general dependencies of the magnetobiology effects’ value on HMC characteristics (induction, exposure duration) and the biological parameter under study. The most pronounced effects were found at the cellular level compared to the organismal level. Gene expression and protein activity appeared to be the most sensitive to HMC among the molecular cellular processes. The nervous system was found to be the most sensitive in the case of the organism level. The review may be of interest to biologists, physicians, physicists, and specialists in interdisciplinary fields.

## 1. Introduction

The relatively strong magnetic field of the Earth (geomagnetic field) is a phenomenon for the group of inner planets of the Solar system [1,2]. It is believed that the presence of a magnetic field plays a key role in providing conditions for the development of life on Earth, along with the presence of water, an atmosphere with an ozone layer, and an optimal distance to the Sun [3]. The geomagnetic field is a global vector field with an induction of 25–65 μT (0.25–0.65 G), depending on the proximity to the Earth’s magnetic poles [4]. Its presence is determined, on the one hand, by the rotation of the Earth’s iron core (Geodynamo), and on the other hand, by the interaction between the solar wind and the Earth’s ionosphere [5,6]. The Earth’s magnetic field consists of several components, the main role among which is played by the main (constant) field. Variations of the geomagnetic field compared to constant components are insignificant (usually up to 1–3%) and are caused by electric current systems in the Earth’s ionosphere [7,8].

There is practically no magnetic field in interplanetary space. Its induction varies between 2 and 8 nT with an average value of ~6.6 nT [9]. The magnetic field in the low-Earth orbit (408 km) is ~15–50 μT [10]. A magnetic field with an induction from 300 nT to 5 μT, according to research data, corresponds to the magnetic field of Mars [11]. The Moon’s magnetic field is even weaker, and its induction does not exceed 300 nT [12,13].

The geomagnetic field performs several functions that ensure the presence of life on Earth. First, it protects the atmosphere from the loss of light elements due to the solar wind [14]. Without the geomagnetic field, the Earth’s atmosphere would be significantly depleted in oxygen and hydrogen and would probably resemble the atmospheres of Mars or Venus [15,16]. In addition, the solar wind initiates radical reactions in the atmosphere, leading to the formation of free radicals of nitrogen and molecular oxygen that react with ozone and contribute to the destruction of the ozone layer [17]. Without the geomagnetic field, virtually the entire ozone layer would be destroyed as a result of these free radical reactions.

Secondly, the geomagnetic field protects the atmosphere from cosmic radiation, consisting of high-energy particles (89% protons, 10% α-particles, and 1% other heavy particles) [14]. Cosmic radiation can increase air ionization, change air flows, and increase the formation of ice crystals in clouds. The latter can significantly increase the reflection of sunlight by the atmosphere and cause cold snaps. Modeling showed that in the absence of the geomagnetic field, cosmic radiation significantly destabilized and cooled the Earth’s climate [3]. It is possible that the presence of the geomagnetic field was a key condition for the onset of abiogenesis due to the “regulation” of doses of solar wind and cosmic radiation in the early stages of the Earth’s existence [18].

Thirdly, the geomagnetic field has a direct impact on living organisms. The most obvious “application” of the geomagnetic field is the orientation of organisms in space with the help of specialized structures: ordered cryptochromes in the bird retina, magnetic nanoparticles distributed in tissues, and magnetosomes in bacteria [19,20,21,22]. It is noteworthy that magnetosomes and magnetotaxis are found in both eukaryotes (migratory songbirds, ungulates) and prokaryotes (iron-containing bacteria of the *Magnetospirillum* species) [20,21,22,23,24,25,26]. The reduction in daily oxygen production by *Elodea* plants was observed under conditions of high geomagnetic activity [27].

**Figure 1:**
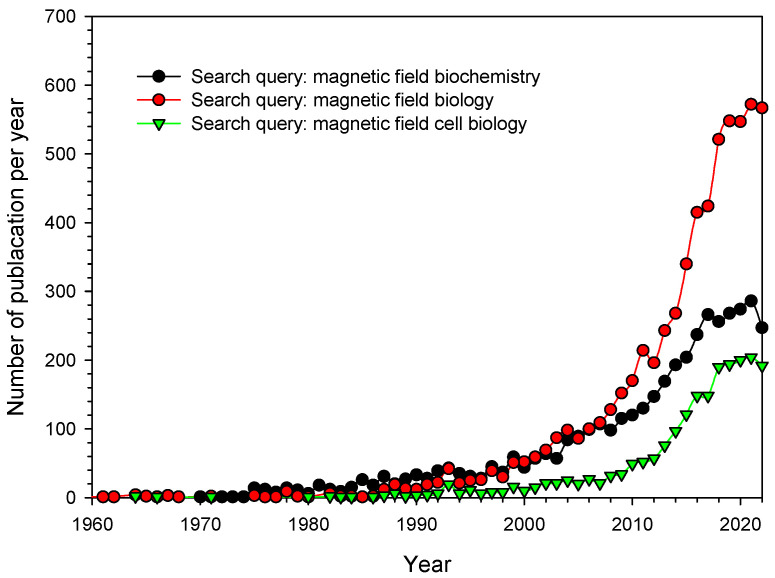
Dynamics of global publishing activity by keywords: magnetic field + biology, magnetic field + cell biology, magnetic field + biochemistry (according to https://pubmed.ncbi.nlm.nih.gov/, accessed on 2 October 2023).

The study of the influence of a magnetic field on living organisms is an important fundamental and applied issue of modern science. The fundamental importance lies in expanding the understanding of the processes of the origin of life on our planet, predicting and searching for potentially habitable planets. The applied significance lies in understanding the influence of long-term exposure to altered magnetic conditions on living organisms, in particular, the effect of hypomagnetic conditions on humans. This is undoubtedly an important aspect of the success of space missions. The relevance of the problem is evidenced by the dynamics of the number of publications on this topic (Figure 1). It is noteworthy that a particularly rapid growth of publications has been noted in the last decade, which indicates an increase in the interest of the world community in this issue. Some decrease in the number of publications this year is probably because the year is not over yet.

In the present review, we attempted to search for relationships between the quantitative characteristics of HMC and the magnitude of the effects described in the literature. We understand that the quality of publications varies considerably in this field of science. Therefore, we selected the following as inclusion criteria: the presence of adequate Sham control and the presence of a description of the type of installation, a description capable of validating the magnetic field induction values during the experiment, and statistical processing of the results. If a paper failed to meet any of these criteria, it was excluded from the analysis.

A recent review aimed to establish correlations between HMC parameters (induction and duration), the method of HMC generation [28], and the magnitude of biological effects. Our analysis is expanded in this study through the examination of MHC effects at further cellular-level parameters. Additionally, we estimated the impact of both HMC validation features and statistical analysis methods.

## 2. Experimental Approaches

**Figure 2:**
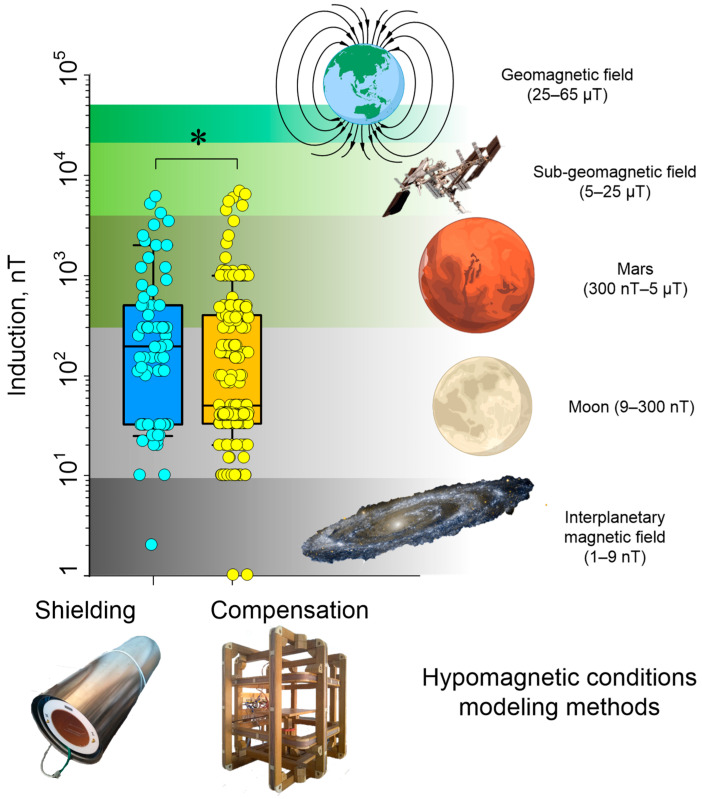
The value of magnetic field induction when modeling HMC (references in Table 1). Each point is the value of magnetic field induction indicated in the literature. The color indicates the method for simulating HMC: orange—compensation using Helmholtz coils, blue—shielding using soft magnetic materials. The induction ranges of the magnetic fields of the Earth and objects in space closest to the Earth are shown using shaded areas. *—*p* < 0.05, Mann–Whitney Rank Sum Test. A total number of analyzed experimental points is 350. The results are shown as medians (box centers) with percentiles 25% and 50% (box bottom and top) and percentiles 10% and 90% (bars).

The effects of hypomagnetic conditions (HMCs) on living systems are diverse and multidirectional (Table 1). The effects of HMC have been studied on animals, plants, and bacteria [29,30,31,32,33], and affect different levels of life organization: from organismal to molecular [34,35]. One of the fundamental and pioneering works in this area is the experiments of Beisher’s group to study the influence of HMC on a person’s spatial orientation in space [36]. To model HMC, one of two approaches is usually used (Figure 2).

The first approach is passive shielding of the geomagnetic field using soft magnetic materials: permalloy (Fe, 45–82% Ni alloy), AMAG alloy, and μ-metal [30,37,38]. Due to the high cost of these alloys, as a rule, experimental chambers made of small-sized soft magnetic materials are used. The working volume of such chambers ranges from ~1 to 125 dm^3^ [39,40]. The second approach is the use of active compensation of the geomagnetic field [36,41]. For this, a system of Helmholtz coils is used, usually three pairs, oriented along three orthogonal axes [35,40]. However, this can also be a single-axis option. In this case, the system axis is directed collinear to the geomagnetic field vector [42,43,44]. The coils generate a magnetic field oppositely directed relative to the geomagnetic field lines and close in induction values to the geomagnetic field induction. Thus, the resulting magnetic field in the internal section of the installation becomes “near zero”. The volume of space with a stable hypomagnetic field, in this case, depends on the size of the coils and, as a rule, ranges from 10 cm × 10 cm × 10 cm to 50 cm × 50 cm × 50 cm [32,34,43,45]. Particularly large installations used for research on humans can have a working volume of up to ~3 m^3^ [43].

Often, during experiments, the local magnetic field changes. These changes vary depending on the geomagnetic situation (changes up to several hundred nT) and urban noise (units of μT) [46]. Therefore, especially when conducting long-term studies in a laboratory within the city, there is a need to organize feedback so that the level of minimum magnetic field induction remains at the required level. Fluxgate magnetic field sensors usually act as a feedback link for assessing the magnetic field induction inside the installation [47,48]. The magnetic field induction, when shielding the geomagnetic field, can be reduced by 10^3^–10^4^ times, and can reach values of <200 nT [49,50] (Figure 2). Compensation systems based on Helmholtz coils have comparable efficiency and allow compensation to be achieved down to resulting field induction values of up to 10 nT or below [44,51]. Recently, Helmholtz coils were used in most of the works. The magnetic field induction, in this case, was lower than in work with soft magnetic materials’ shielding chamber (Figure 2). Compensation of the variable magnetic field component in systems based on Helmholtz coils has its limitations. Thus, in the installation described in the works, a fluxgate sensor (high-sensitive three-axial sensor FL3-100, produced by Stefan Mayer Instruments, Dinslaken, Germany) is used [52,53]. Since the sensor bandwidth is limited to the frequency range 0 to 2 kHz (−3 dB), compensation of variable magnetic fields was only possible for low-frequency magnetic fields. At a frequency of 1 Hz, the compensation was 10^3^ times, at 50 Hz, 8–10 times, and at a frequency of 500 Hz, compensation no longer occurred.

In general, Helmholtz systems are cheaper to manufacture, can adjust to the exact value of the magnetic field induction in the working area, and also allow, if necessary, for providing a superposition of a constant hypomagnetic field and an alternating magnetic field with given amplitude–frequency characteristics [52,53,54,55,56]. The growing popularity of Helmholtz systems is also due to the gradual increase in the quality and availability of electronic feedback components. All this makes Helmholtz systems more attractive for biological experiments (~65% of analyzed works), compared to chambers made of soft magnetic materials (~35% of analyzed works) (see Table 1).

**Table 1:** Examples of the effects of HMC on animals (organ and organism level).

№	Biological Object	Characteristics	Effect, %	Magnetic Flux Density	Time	N	Statistic	Validation	Experimental Setup	Size or Volume	SJR	Ref.
1	Human, men and women, average age of 45 ± 18 years	Heartbeat rate (≥40 years) Heartbeat rate (<40 years) Diastolic blood pressure (under 40 years) Capillary blood flow rate	−20% +10–15% −4–5% +22–23%	10 nT >> >> >>	120 min >> >> >>	32 >> >> >>	ANOVA, F-test	Magnetometer, 3-axis, spatial distribution, HMF variation < 10 nT GMF: ~48 μT, meteorological data were used to choose assay days	Helmholtz coils (3-axis)	2.6 m^3^	0.42 (Q2)	[44]
2	Rat *Rattus norvegicus* adult	Number of erythrocytes (RBC), Hematocrit (EPV), Erythrocyte volume (MCV), Hemolysis	+12% +7% −10% −85%	0.192 μT >> >> >>	1–4 days >> >> >>	3 >> >> >>	Student’s paired *t*-test	Magnetometer, 3-axis, 1 point	Shielding chamber from amorphous magnetic material AMAG-172	-	-	[37]
3	Zebrafish *Danio renio* wild type (AB strain), embryos	Viability Heartbeat rate	−10% +5%	<300 nT >>	120 h >>	200 >>	Shapiro–Wilk W-test or Kolmogorov–Smirnov test, Levene’s test, *t*-test, Cosinor analysis (for circadian rhythms)	Magnetometer 3-axis spatial distribution GMF: 51.7 μT AFM: 50 Hz, < 15 nT without harmonics	Helmholtz coils (3-axis)	Ø 50 cm	1.03 (Q1)	[48]
4	Human men (24–53 years) and women (26–49 years)	Higher nervous activity: test for matching the meaning of a word and its color: lead time error rate Letter recognition test: lead time error rate	+10% +15% +5% +150%	<0.4 μT >> >> >>	1 h 17 min >> >> >>	40 >> >> >>	Multivariate analysis of variance (MANOVA)	Magnetometer, 3-axis, spatial distribution, variation < 0.2 μT GMF: ~41 μT AMF variations complicated	Helmholtz coils	~3 m^3^	0.4 (Q3)	[42]
5	Rat, *Rattus norvegicus* line Wistar	Open field testing: horizontal component, vertical component, general physical activity Power of EEG rhythms: Theta Alpha Beta Gamma	−20% −30% −50% −50% −50% −50% −50%	50 nT >> >> >> >> >> >>	21 days >> >> >> >> >> >>	12 >> >> >> >> >> >>	Wilcoxon signed-rank test, Kolmogorov–Smirnov test	Magnetometer, 3-axis, 1 point, HMF variation: < 50 nT	Helmholtz coils (2-axis)	Ø 50 cm	-	[57]
6	Rat, *Rattus norvegicus* line Wistar	Number of aggression acts (day) Number of aggression acts (night)	+130% +17 times	50–150 nT >>	21 days >>	12 >>	Wilcoxon signed-rank test, Kolmogorov–Smirnov test	Magnetometer, 3-axis, 1 point, HMF variation: < 50 nT	Helmholtz coils	Ø 50 cm	-	[58]
7	Golden hamster *Ochrotomys nuttalli* adults	Proportion of noradrenergic neurons in areas A3 and A7 of the brainstem	−29% −35%	22 nT >>	60 180 days	5 >>	One-way ANOVA or Student’s *t*-test	Magnetometer 3-axis spatial distribution: 0.022–2.8 μT	Permalloy chamber	70 cm × 70 cm × 90 cm	0.42 (Q3)	[59]
8	Mice (*M. musculus*) C57BL/6 J adults, 8–10 weeks	Behavioral tests: Freezing in context test Freezing in cue test	−15% −12%	170 nT >>	8 weeks >>	10 >>	One-way or two-way ANOVA or Student’s *t*-test	Magnetometer, 3-axis, spatial distribution, ambient magnetic fields, noise and light were measured. SMF in incubator: 39.4 ± 3.6 μT. AMF: 50 Hz Bt PSD1/2 2.37 nT/√Hz	Helmholtz coils (3-axis)	Ø 50 cm	5.12 (Q1)	[60]
9	Mice, C57BL/6J, 7 weeks old	Open field behavior test: percent time spent in the center, total traveled distances, time spent exploring the novel location, time spent exploring a novel object	−80% 0% −30% −30%	31.9 nT >> >> >>	8 weeks >> >> >>	10 >> >> >>	Double-blind study, unpaired Student’s *t*-test	Magnetometer 3-axis 1 point, time distribution, HMF variation: < 4.5 nT GMF: ~55 μT temperature, illumination, and relative humidity equal in all conditions	Helmholtz coils (3-axis)	2 m × 2 m × 2 m	1.15 (Q1)	[61]
10	Chicken *Gallus gallus domesticus* incubated in hypomagnetic conditions, eggs and chicks hatched from them	Retained curve in one-trial passive avoidance task (OTPAT) Temporary mean memory test Long-term memory test	−68.4% −74.8%	354 nT >>	21 days >>	10 >>	One-way ANOVA	Magnetometer, 1-axis, 1 point HMF variation: < 254 nT	Helmholtz coils (3-axis)	Ø 120 cm	1.45	[62]
11	Fruit fly *Drosophila melanogaster* imago, females 3–4-diurnal Prussian wild type (10–19 successive generations)	Performance index (PI) of operant visual learning and memory (L/M) formation of flies	−65%	100–680 nT	40–80 days	445	One-way ANOVA	Magnetometer, 1-axis, spatial distribution, GMF: 52.21 μT	Helmholtz coils (3-axis)	50 cm × 50 cm × 50 cm	0.8 (Q2)	[63]
12	Brown planthopper, *Nilaparvata lugens* males and females, imago	Direction of movement in food (decrease transition to random movement)	−100%	~500 nT	24–48 h	500	Student’s *t*-test	Magnetometer, 3-axis, spatial distribution (homogeneity HMF at Ø 150 mm) GMF: 52.5 ± 0.8 μT	Helmholtz coils (3-axis)	Ø 15 cm	0.7 (Q1)	[64]
13	Oriental armyworm, *Mythimna separata*, adults, males and females	Flight spatial orientation	−100%	500 nT	20 s	9	Rayleigh’s test, Watson–Williams test	Magnetometer, 3-axis, 3D map, HMF variation: < 4%	Helmholtz coils	Ø 120 cm	0.82 (Q1)	[65]
14	Black Garden Ant (*Lasius niger*)	Behavior: Time to reach food, Time to return to the nest, Mistakes to reach food	+200% +40% +300%	~40 nT >> >>	14 days >> >>	1000 >> >>	Kolmogorov–Smirnov test, one-way ANOVA, Tukey’s post hoc test	Magnetometer, 3-axis, spatial distribution, HMF variation: < 6 µT GMF: ~42 µT GMF variation: <20 nT	Helmholtz coils (3-axis)	Ø 128 cm	1.15 (Q1)	[66]
15	Brown planthopper, *S. furcifera*, males and females, imago	Positive phototaxis Speed, duration, and range of flight Body weight	−20% −40% −8%	~477 nT >> >>	1–5 days >> >>	40 >> >>	One-way or two-way ANOVA	Magnetometer, 1-axis, spatial distribution (0–1.06 μT) GMF: ~50 μT	Helmholtz coils (3-axis)	Ø 30 cm	0.74 (Q1)	[45]
16	Rat *(Rattus norvegicus*) Wistar line, females and males	Concentration of Fe, Mn, Co, Ni, Cr, Cu in hair	−5–40% (depending on the element and sex of the animal)	<20 nT	7 months	8	One-way ANOVA	Magnetometer 1-axis 1 point	Chamber from steel type S235JRG2	~1 m × 1 m × 1 m	0.94 (Q1)	[29]
17	Fishes, 0–1 year, *Carassius carassius*, *Rutilus rutilus*, *Cyprinus carpio*, snail *Limnaea stagnalis*, planktonic crustaceans, *Daphnia magna* (imago)	The concentration of Fe, Mn, Co, Ni, Cr, Cu in the brain, skeletal muscles (fishes), or all organisms (daphnia)	−50 % (depending on the element, organ, and species)	<10 nT	1 h	7	Mann–Whitney test	Magnetometer 1-axis 1 point GMF: 51.7 μT	Helmholtz coils (3-, 1-axis)	Ø 50 cm	0.31 (Q3)	[67]
18	Brown planthopper, *Nilaparvata lugens* migrating adults, eggs	Body weight of hatched insects Body weight of 5th instar nymphs Feeding of 5th instar nymphs Glucose content in 5th instar nymphs	15% −35% −35% +20% −15%	480 nT >> >> >> >>	48 h >> >> >> >>	20 >> >> >> >>	Shapiro–Wilk test, Levene’s test, one-way ANOVA, or Mann–Witney U-test	Magnetometer, 1-axis, spatial distribution, HMF variation: < 5% GMF: ~50 μT	Shielding chamber from μ-metal alloy and Helmholtz coils (3-axis)	Ø 30 cm	0.94 (Q1)	[68]
19	Rat *Rattus norvegicus* line Sprague Dawley 250–270 g	Body weight Strength characteristics of bones: Ultimate Power Hardness factor Elastic modulus Density Weight Number of trabeculae Degree of bone anisotropy concentration of receptor activator of nuclear factor-kB ligand (RANKL) in bone tissue Serum: Concentrations of bALP, DPD, and GCs	−17% −18% +18% +17% −18% −15% +50% −25% −75% +35%	<300 nT >> >> >> >> >> >> >> >> >>	28 days >> >> >> >> >> >> >> >> >>	30 >> >> >> >> >> >> >> >> >>	One-way or two-way ANOVA	Magnetometer, 1-axis, 1 point GMF: ~50 µT, illumination and ventilation conditions as HMF and GMF were equal	Shielding chamber (aluminum/permalloy/silicone/iron)	1.86 m × 1.66 m × 1.5 m	Rat (*Rattus norvegicus*) line Sprague Dawley, 250–270 g	[69]
20	Mice, males C57BL/6 hindlimb suspension model	Bone mineral content, Ultimate bending moment, Ultimate stress, Bone volume fraction, Trabecular separation, Connectivity density, Osteoblast number, Osteoclast number, Osteoclast surface, Bone eroded surface, Serum levels of tartrate- resistant acid phosphatase (bone resorption marker) Serum iron, Ferritin level Total iron content: liver, spleen Bone iron, Bone marrow iron	−20% −15% −15% −40% +15% −40% −40% +30% +15% +30% +20% +30% +20% +20% +35% +20% +20%	<300 nT >> >> >> >> >> >> >> >> >> >> >> >> >> >> >> >>	4 weeks >> >> >> >> >> >> >> >> >> >> >> >> >> >> >> >>	6 >> >> >> >> >> >> >> >> >> >> >> >> >> >> >> >>	Two-way ANOVA, Sidak’s post hoc test	Magnetometer, spatial distribution, AMF in control incubator 50 Hz ~1 μT AMF in an experimental incubator 50 Hz, < 12 nT GMF: ~45 μT	Permalloy chamber	550 m × 420 m × 420 m	1.13 (Q1)	[70]
21	Mouse *M. musculus* line NMRIz, pregnant females, embryos 3 days after fertilization	Birth rate, Number of implanted embryos, Histological abnormalities, resorption	−30% −30% +Qualitatively	<200 nT >> >>	12 days >> >>	5 >> >>	Student’s *t*-test	Magnetometer, 1-axis, 1 point, GMF: ~40 μT	Permalloy chamber	-	0.4 (Q3)	[71]
22	Brown planthopper *S. furcifera* eggs and nymphs	Body weight (2 days old): Female, Male Positive chemotaxis: Females (5 days old), Males (2 days old), Males (5 days old) Flight speed (2 days old): Females, Males Flight duration: Female, Male Flight distance: Female, Male	−5% −10% +40% +30% +30% +30% −20% −80% +40% −60% N/A	477 nT >> >> >> >> >> >> >> >> >> >>	2000 h >> >> >> >> >> >> >> >> >> >>	40 >> 115 >> >> 23 46 23 46 23 46	Two-way ANOVA, MANOVA, Shapiro–Wilk test (normality), chi-square test (two-tailed) with Yates’s correction, Student’s *t*-test	Magnetometer, 3-axis, one point, HMF variation: < 25 nT GMF: ~52 μT, temperature variation: < 0.1 °C	Helmholtz coils	Ø 120 cm	1.04 (Q1)	[72]
23	Oriental armyworm; *Mythimna separata* eggs, larvae, pupae, and imago (females and males)	Duration of development stages: larval doll imago (males) Pupa mass Number of eggs laid by one female	+5% +2% +5% −20% −5% −45%	<500 nT >> >> >> >> >>	12 h >> >> >> >> >>	300 >> >> >> >> >>	One-way or two-way ANOVA	Magnetometer, 1-axis, 1 point, time distribution, HMF variation: < 500 nT	Helmholtz coils	Ø 50 mm	0.94 (Q1)	[73]
24	Crustaceans, *Daphnia magna* *Daphnia carinata* newborns and adults	Newborn sizes Adult sizes Life length	−15% −5% −5%	<15 nT >> >>	24 h >> >>	30 >> >>	Kolmogorov–Smirnov test, Levene’s test (homoscedasticity), one-way analysis of variance (ANOVA), Dunnett’s post hoc test	Magnetometer, 3-axis, spatial distribution AFM: 50 Hz < 12 nT GMF 51.7 mT	Helmholtz coils (3-axis)	Ø 50 cm	0.4 (Q3)	[74]
25	Human men and women (<40 years)	Pupil diameter	+1.6%	300–600 nT	40 min	40	One-way ANOVA	Magnetometer, 3-axis, spatial distribution, variation: < 0.4 μT GMF: ~41 μT AMF variations complicated	Helmholtz coils	1 m × 1 m × 1.5 m	-	[75]
26	Tardigrades *(Paramacrobiotus experimentalis)* females and males of different age	Proportions of active animals	−10%	<250 nT	7 days	45	two-way ANOVA, Tukey post hoc test	Magnetometer 1-axis 1 point GMF: ~50 μT	μ-Metal shielding chamber (approximately 77% nickel, 16% iron, 5% copper, and 2% molybdenum)	18.5 cm × 12 cm × 33 cm	1.03 (Q1)	[76]
27	*Helix albescens* large common snail	Duration of circadian rhythms	−17% +19%	0.5–2 µT >>	3 days 21 days	20 >>	Fourier transformation, Student’s *t*-test (normality tested)	Magnetometer, 1-axis, 1 point, spectral density of magnetic noise: < 10 nT/Hz	Room covered with Dynamo iron leaves	2 m × 3 m × 2 m	1.07 (Q1)	[77]
28	Tardigrades Echiniscus testudo and *Milnesium inceptum*	Mortality rate: (1) dehydrated (2) during dehydration (3) returning to active life after dehydration	+45% +80% +200%	<25 nT >> >>	21 days >> >>	100 >> >>	One-way ANOVA, Tukey test as a post hoc test, or Student’s *t*-test with the Cochran–Cox adjustment	Magnetometer, 1-axis, 1 point GMF: ~50 GMF	Shielding chamber amorphous magnet (μ-metal)	18.5 cm × 12 cm × 33 cm	0.7 (Q1)	[38]

N/A—effect was not observed. ANOVA—analysis of variance, spatial distribution—the authors indicate an assessment of the spatial distribution of the magnetic field; numerical values of variation are given, 3D map—a detailed image of the spatial distribution of magnetic field induction is given.

## 3. Effects of HMC on Living Objects

### 3.1. Effects of HMC on Animals (Organ and Organism Level)

**Figure 3:**
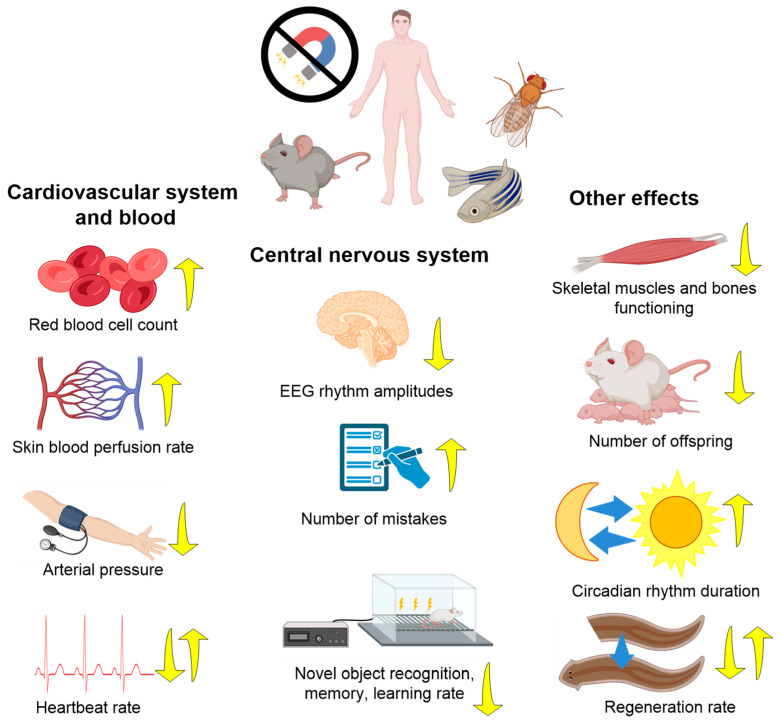
Main directions of effects of HMC on animals. The arrows indicate the direction of the effect: decrease (down) or increase (up) of the parameter. The center shows examples of the most commonly studied model organisms: humans, rodents, insects, and aquatic animals (references in Table 1).

Studies of the influence of HMC are most interesting for solving questions about the planning of long-term space expeditions; therefore, a significant part of the work was carried out on animals. The effects of HMC have been studied both on the body as a whole and individual systems, nervous, circulatory, musculoskeletal, reproductive, etc., on processes at the cellular and molecular levels. For the convenience of the reader, we will begin with the effects of HMC at the organismal level (Table 1, Figure 3).

#### 3.1.1. Nervous System

The effect of the hypomagnetic field on behavior can also be attributed to the organismal level. The influence of HMC on the functioning of the central nervous system manifests itself in the form of several effects described below. HMCs have been shown to reduce human cognitive abilities. A 40-minute stay in hypomagnetic conditions reduces the ability to solve problems such as letter recognition (Shepard test), determine the relationship between “color and its name” (Stroop test), and perform other cognitive tests [43]. The increasing errors and decreasing rate of responses of HMC compared to the control (geomagnetic field) in some tests reached 5% [42,43]. It is worth noting that several papers, including pioneering ones, did not find any effects of HMC on the results of the tests of spatial orientation, body position, and spatial memory [36].

Experiments with animals showed that mice raised in HMC showed a decrease in “freezing time” to a fearful stimulus encountered earlier, indicating a disruption in the processes of memory formation, evaluation of new environments, and reproduction of previously experienced experiences [60]. Similar results were obtained in chicks hatched from eggs incubated in HMC [62]. HMC caused an increase in aggression in rats and decreased the number of opioidergic neurons in the brain [58]. It is noteworthy that another study in rats found no effects of HMC when tested in the “open field”, but at the same time, changes in the electroencephalography (EEG) were observed [57].

Consequently, the effects of HMC depend on the animal species and the parameter being assessed; in some species, these effects can be compensated for, at least by their behavioral manifestations [57]. In the case of invertebrates (*Drosophila melanogaster*), HMCs also disrupt the “learning” processes by more than two times [63]. The HMC also shows a deterioration in search behavior and spatial memory of ants. These changes were associated with biochemical changes in the nervous system [66]. HMCs enhanced positive phototaxis and loss of directional movement in foraging in insects [45]. Using snails as an example, a prolongation of circadian rhythms by one and a half times compared with the control was shown [77]. This effect does not directly relate to HMC, but allows us to estimate the contribution of induced weak fields. Such changes in behavior may be the consequence of significant changes in the functioning of the central nervous system. The powers of alpha, beta, gamma, and theta rhythms decreased in the EEG in rats under the influence of HMC [57]. A more pronounced effect of HMC was found in the brain of Ansell’s mole-rats (*Fukomys anselli*). These animals lack vision and orientation along magnetic field lines, playing an important role in their behavior. HMC, even with short incubations, significantly changed the expression of c-Fos (a protein that regulates neuronal development) in different parts of the brain [78].

#### 3.1.2. Cardiovascular System and Immunity

In humans, changes affect both the macrocirculatory system and microcirculation. For macrocirculation, a decrease in heart rate and diastolic pressure under the influence of HMC is detected [44]. For microcirculation, the opposite effect is found—an increase in the speed of blood flow in the capillaries [79]. An increase in capillary blood flow velocity may be the cause of a compensatory decrease in heart rate [79]. The effects of HMC can be explained by changes in the modulating influence of the parasympathetic division of the autonomic nervous system [80]. HMCs also change the characteristics and composition of the blood. In HMC rats, an increase in erythrocyte count and hematocrit, a decrease in erythrocyte volume, and a significant decrease in erythrocyte hemolysis were observed after 204 h of incubation [37]. The latter result may open up new prospects for the use of hypomagnetic fields for storing donor blood [37]. However, in another study, an increase in hemolysis of human blood was observed in HMC [81]. The differences in the data may be explained by different degrees of attenuation of the geomagnetic field: at ~200 nT, a reduction in hemolysis was observed, and at ~100 nT, hemolysis was enhanced [37,81]. On the part of the immune system, weakening of the PMA- and fMLF-induced “respiratory burst” of peritoneal neutrophils was found [82]. At the same time, the concentration of granulocytes in the blood increases [83]. HMCs also affect the functioning of the cardiovascular, hematopoietic, and immune systems. In particular, HMC caused an increase in heart rate in *Danio renio* fish embryos [48]. In mice, changes in the tissue and cellular structure of the myocardium occur [84].

#### 3.1.3. Musculoskeletal System, Metabolism, and Other Effects

The effect of HMC on the musculoskeletal system is the deterioration of the functional state of skeletal muscles, the mechanism of which may be a violation of muscle metabolism [85]. Metabolic changes are expressed in a decrease in the concentration of citric acid and ATP, an increase in the ATP/ADP ratio, as well as a decrease in consumption regarding a load of glucose and glycogen [85,86]. In experiments on rats with the combination of hypomagnetic and microgravity conditions, HMCs have been found to have a leading contribution to the disruption of bone structure and an increase in their fragility [69]. In another study, HMC did not affect bone structure and mechanical properties. However, it did accelerate bone destruction processes in a hindlimb load reduction model. The combination of the HMC and hindlimb suspension model demonstrated considerable reductions in bone mineralization, bone volume fraction, and connectivity density compared to the hindlimb suspension model alone. Structural modifications have hurt the biomechanical characteristics of bone, namely the ultimate bending moment and ultimate stress [70]. Published data indicate that not only microgravity, but also a weakening magnetic field, is a risk factor for the development of muscle disorders in astronauts.

HMCs influence the reproduction and embryonic development of animals. Using the example of insects, it has been shown that HMCs reduce the number of eggs produced by one female and increase the time of development of larvae to adults [45]. The quality and motility of gametes (fruit fly *Drosophila melanogaster*) were also reduced in HMC [51]. For daphnia, a decrease in the size of newborn individuals, as well as the life span of adults, has also been shown [74]. For vertebrates, the teratogenic effect of HMC has also been discovered. In frogs, a three-fold increase in malformations was observed [40]. In laboratory mice, an HMC-induced decrease in the birth rate by a third has been described, due to disruption of the processes of embryo implantation, embryo resorption, and disruption of the integrity of the endometrium [71]. In the case of studies of embryogenesis, not only the presence of a hypomagnetic field is important, but also the start time of exposure [87]. It is noteworthy that HMC does not affect (or only slightly affects) the functioning of the digestive system. For example, HMC does not alter water and food intake in mice [83]. HMUs have been shown to reduce the viability of tardigrades (*Echiniscus testudo* and *Milnesium inceptumto)* after dehydration [38]. The oriental armyworm (*Mythimna separate*) had a complete loss of flight spatial orientation at HMC (500 nT, 20 s) [65]. Prolonged exposure of brown planthopper eggs and nymphs leads to enhanced positive phototaxis in adults and causes a significant alteration in flight characteristics such as duration, range, and speed [72].

### 3.2. Effects of HMC on Plants

**Figure 4:**
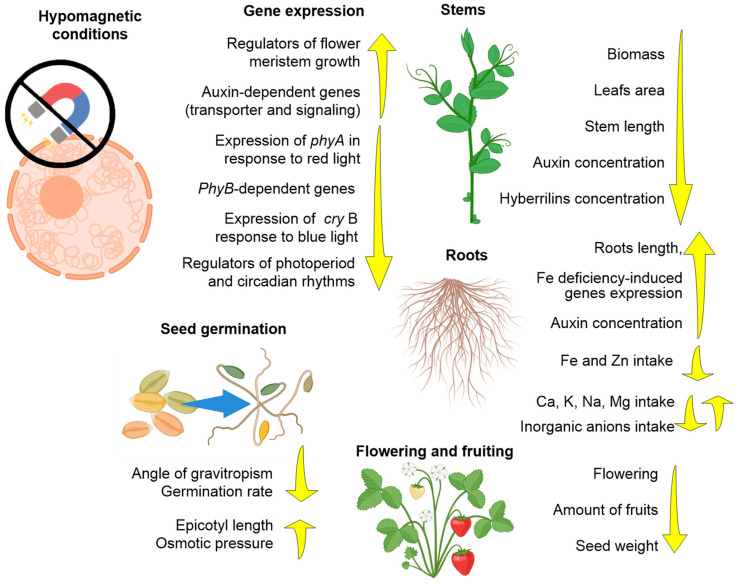
The main directions of the effects of hypomagnetic conditions on the morphology and physiology of plants. Arrows indicate the direction of the effect: decrease (down) or increase (up) in the parameter (references in Table 1).

The effects of HMC on plants include systemic reactions of the whole organism, and the effects on individual organs or molecular targets (Table 2, Figure 4).

Soybean and Arabidopsis are most often used as model plants [88,89]. With HMC, seed germination and the growth rate of germinal roots decrease [89]. The accumulation of biomass (both dry and “wet”), the leaf area index, and the number of seeds per plant are significantly reduced in HMC [89,90]. The time of seed germination, flowering, and fruiting is prolonged under HMC [89,90]. It is noteworthy that returning plants to geomagnetic conditions from HMC conditions restores these parameters [90]. A constant hypomagnetic field causes weakening of the gravitropism of soybean seedlings [88]. At the molecular cellular level, a decrease in the consumption of Fe and Zn by roots and the launch of signaling cascades in response to iron deficiency were detected [91]. HMCs demonstrated a complex effect on plant consumption of both cations (NH_3_^+^, K^+^, Ca^2+^, and Mg^2+^) and anions (Cl^−^, SO_4_^2−^, NO_3_^−^, and PO4^−3^) via an increase in the expression of Ca^2+^ and Mg^2^ cation and Cl^−^, SO_4_^2−^, NO_3_^−^, and PO4^−3^ anion transporter proteins [92]. In addition, HMCs reduce the expression of regulators of circadian rhythms and floral meristem growth [90]. Using peas as an example, HMCs cause an increase in osmotic pressure in the roots of seedlings [93]. It has been shown that HMCs cause an increase in the concentration of the stress hormone gibberellin in plants and the launch of stress-activated signaling cascades [31]. An unobvious effect of HMC is a significant (two-fold) increase in the expression of proteins that regulate the response to light (cryptochrome A and phytochrome A) and a decrease in the expression of phytochrome B [94]. Activation of the phytochrome system enhances auxin synthesis in roots and reduces it in above-ground parts, changes the regulation of auxin-induced genes, enhances root growth, and inhibits stem growth; as a result, plants acquire rosette morphology [95,96]. The authors suggest that plant phytochrome signaling systems are involved in the response of plants to HMC [94]. Other studies have shown that HMC causes a redistribution of the concentrations of photosynthetic pigments in lima bean leaves, and also reduces the formation of ROS (H_2_O_2_) due to an increase in the expression of antioxidant enzymes [97]. These data may shed light on possible complications with the cultivation of plants onboard space stations of the future and possible ways to overcome them.

**Table 2:** Examples of the effects of HMC plants.

№	Biological Object	Characteristics	Effect, %	Magnetic Flux Density	Time	N	Statistic	Validation	Experimental Setup	Size or Volume	SJR	Ref.
1	*Arabidopsis thaliana* seedlings, WT and *spl7-*KO	Fe uptake by roots, Zn uptake by roots, Expression of Fe-deficiency-induced genes in roots: *IRT1*, *AHA2*, *FIT*, *ILR*, *bHLH38*, *bHLH39*, *3*, *FRO2*, *Spl7* knockout or Fe supplementation alters hypomagnetic condition effects	−2 times −2 times +2–10 times −2–3 times	~40 μT >> >> >>	96 h >> >> >>	4 >> 3 >>	One-way ANOVA	Magnetometer, 3-axis, spatial distribution, GMF: 41–43 μT	Helmholtz coils (1-axis)	-	1.23 (Q1)	[91]
2	*Arabidopsis thaliana Landsberg erecta*, wild type, seedlings	Hypocotyl lengths: blue light, darkness	−3% +6%	~10 nT >>	72 h >>	30 >>	Paired *t*-test	Magnetometer, 1-axis, 1 point SMD variation: < 10 μT GMF: ~50 mT	Helmholtz coils (1-axis)	Ø 22 cm	1.2 (Q1)	[98]
3	*Arabidopsis thaliana*, wild type and *spl7*, *amiFRO5*, and *amiFRO4/5* mutant lines	Fe concentration: control S index: S deficit Shoot area: control, Fe deficit Root length: control, Fe deficit, S deficit, Fe and S deficit Gene expression (part): *AHA* (Fe deficit), *FRO* (Fe and S deficit), *PYE* (Fe deficit), *bHLH38* (Fe and S deficit)	−25% −20% −5% −5% −10% −10% −10% −10% −55% +45% −50% +50%	42 nT >> >> >> >> >> >> >> >> >> >> >>	7 days >> >> >> >> >> >> >> >> >> >> >>	3 >> >> >> >> >> >> >> >> >> >> >>	Two-way ANOVA, Tukey’s post hoc test	Magnetometer, 3-axis, time distribution, variation: < 2nT, GMF: 41–43 μT	Helmholtz coil (3-axis)	Ø 128 cm	1.15 (Q1)	[99]
4	Lima bean (*Phaseolus lunatus*) seeds and seedlings	Tomato leaf density, leaf area, relative water content, the major axis of chloroplast length, total carbohydrate content, total protein content, percentage of leaf carbon, carbon isotope discrimination (δ13C) Concentrations: Chlorophyll a, Chlorophyll b, Chlorophyll a’, Chlorophyll b’, Pheophytin a, Lutein, *Trans*-α-carotene, *cis*-α-carotene, *Trans*-β-carotene, 9-*cis*-β-carotene Protein expression: catalase, ascorbate peroxidase, peroxidase, glutathione reductase, glutathione peroxidase ROS production: peroxide, H_2_O_2_	+50% −30% N/A +20% −20% +10% +5% +30% −20% −20% +250% +100% +100% −40% −30% −25% −75% −40% −25% −2500% +10% −200% +200% +500% −75% −10%	~40 nT >> >> >> >> >> >> >> >> >> >> >> >> >> >> >> >> >> >> >> >> >> >> >> >> >>	96 h >> >> >> >> >> >> >> >> >> >> >> >> >> >> >> >> >> >> >> >> >> >> >> >> >>	3 >> >> >> >> >> >> >> >> >> >> >> >> >> >> >> >> >> >> >> >> >> >> >> >> >>	Paired Student’s *t*-test and Bonferroni post hoc test	Magnetometer, 3-axis, time distribution, variation: < 2 nT GMF: 41.94 μT	Helmholtz coil (3-axis)	Ø 128 cm	1.15 (Q1)	[97]
5	*Arabidopsis thaliana* ecotype *Landsberg erecta*, WT or cry1cry2 mutants	Photosynthesis gene expression: *rbcl* (ribulose 1,5 bisphosphate), *cab4* (chlorophyll a,b binding protein), *pal4* (phenylalanine ammonia lyase), *ef1* (elongation factor-1)	−20% −60% −20% −5%	0.2 μT >> >> >>	120 h >> >> >>	4 >> >> >>	Student’s *t*-test	Magnetometer, 3-axis, spatial distribution, power supplies were separated from the μ-metal cylinder GMF: ~38 μT	Faraday-cage room, Helmholtz coils (2-axis)	5.04 × 2.04 × 2.1 m Ø 18 cm	0.88 (Q1)	[100]
6	*Arabidopsis thaliana* ecotype Col0, seedlings	Expression of circadian rhythm regulator genes: *LHY*, *PRR7*, *GI*	−80% −80% +60%	~40 nT >> >>	7 days >> >>	3 >> >>	Two-way ANOVA, Tukey post hoc test	Magnetometer, 3-axis, spatial distribution, GMF: 40–45 μT	Helmholtz coil (3-axis)	-	0.88 (Q1) >>	[101]
7	*Arabidopsis thaliana* (Col-0), Wt and *cry1cry2*-, *phot1*-, *phyA*-, *and phyAphyB-*deficient mutants, seedlings (1 week)	Changes in cryptochrome expression in response to blue light: Wt, *phyA* mutant Changes in *phyA* (phytochrome A) expression in response to red light Changes in cryptochrome expression in response to red light	+100% −100% −100%	~40 nT >> >>	96 h >> >>	3 >> >>	Kolmogorov–Smirnov test (normality), one-way ANOVA, Tukey, and Bonferroni post hoc tests	Magnetometer, 3-axis, spatial distribution, sample rate: 10 s	Helmholtz coil (3-axis)	-	0.87 (Q1)	[94]
8	Soy *Glycine max* seeds and seedlings	Gravitropism angle, Radicle weight ratio, Germination percentage, Germination rate, A ratio of root length to seed length	−50% +18% N/A −10% +12%	<111 nT >> >> >> >>	1 h >> >> >> >>	10 >> >> >> >>	Two-way ANOVA	Magnetometer 3-axis 1 point Temperature and relative humidity equal in both conditions	Chamber from 12 layers of permalloy sheets, enclosed within an outer aluminum layer	~10 cm × 10 cm × 10 cm	0.6 (Q2)	[88]
9	*Arabidopsis thaliana* ecotype Columbia	Epicotyl length, Adult habitus-acquisition of rosette morphology, Expression of phytochrome B signaling pathway genes: *PHYB*, *CO*, *FT*	+30% qualitatively −40% −40% −50%	<50 nT >> >> >> >>	36 days >> >> >> >>	20 3 >> >>	Student’s *t*-test	Magnetometer, 3-axis, spatial distribution, GMF: ~45 μT	Helmholtz coil (axis)	Ø 88 cm	0.6 (Q2)	[96]
10	*Arabidopsis thaliana* Adult	Biomass (total) Biomass (dry) Flowering time Number of fruits per plant Seed weight per plant Harvest index (ratio between seed weight and total biomass)	−30% −40% +5% −20% −20% −20%	<1 μT >> >> >> >> >>	35 days >> >> >> >> >>	20 >> >> >> >> >>	One-way ANOVA	Magnetometer, 3-axis, 3D map GMF: ~42 μT HMF variation: < 50 nT	Helmholtz coil (3-axis)	Ø 80 cm	0.43 (Q3)	[89]
11	*Arabidopsis thaliana* (Col-0), Wt	Time from germination to flowering, Time from germination to fruiting, Restoration of characteristics above after change in hypomagnetic condition to geomagnetic Leaf area index, Stem length, Expression of clock genes and photoperiod pathway genes, Expression of floral meristem genes, Expression of *GA20ox2*	+20% +15% +100% −15% −30% −1.5–2.2 times −3–5 times −50 times	41 nT >> >> >> >> >> >> >>	15 min >> >> >> >> >> >> >>	15 >> >> >> >> >> >> >>	Kolmogorov–Smirnov test, one-way ANOVA	Magnetometer, 3-axis, time distribution, variation: < 2 nT GMF: 41.94 μT	Helmholtz coil (3-axis)	Ø 128 cm	0.43 (Q3)	[90]
12	*Arabidopsis thaliana* ecotype Columbia Col-4 Adult WT cry1-/cry2-mutants	WT: Expression of GA3ox1, Expression of GA3ox2, Expression of GA3ox3, LFY, SOC1, Gibberrilin concentration cry1-/cry2-mutants: Expression of GA3ox1, Expression of GA3ox2, Expression of GA3ox3, Gibberrilin concentration	−45% −55% −55% −35% −30% ~50% - 0 0 0	<1 μT >> >> >> >> >> >> >> >>	33 days >> >> >> >> >> >> >>	3 >> >> >> >> >> >> >>	One-way ANOVA	Magnetometer, 3-axis, spatial distribution, GMF: ~45 μT	Helmholtz coil (3-axis)	Ø 88 cm	0.42 (Q3)	[31]
13	*Arabidopsis thaliana* ecotype Columbia Col-4 Adult WT cry1-/cry2-mutants	WT: Auxin Levels in leaves, Auxin Levels in roots, Expressions of Auxin Transporter Genes, Expressions of Auxin Signaling Genes cry1-/cry2-mutants: Inhibition of the hypomagnetic field effects	−25% +40% +20% +30% 0	<1 μT >> >> >> >>	33 days >> >> >> >>	3 >> >> >> >>	One-way ANOVA	Magnetometer, 3-axis, spatial distribution, GMF: ~45 μT	Helmholtz coil	Ø 20 cm	0.42 (Q3)	[95]
14	*Arabidopsis thaliana* adult, wild type	Cation content in roots: NH_3_^+^, K^+^, Ca^2+^, Mg^2+^ Gene expression: Ca^2+^-transporting ATPase 11, Mg^2+^ transporter CorA-like protein-related Aniom content in roots: Cl^−^, SO_4_^2−^, NO_3_^−^, PO4^−3^, Gene expression: Cl^−^ channel protein (CLC-A), Cl^−^ channel protein (CLC-C), Cl^−^ channel protein (CLC-G), SO_4−_ transporter (Sultr3;1), NO_3_^−^ transporter (NRT1.6), NO_3_^−^ transporter (NRT2.4), PO_4_^3-^ transporter (PHT1;8)	+25% +5% −15% +5% +50% −5% −15% −10% −50% +80% −10% +40% −90% +30% +8% −15% +10% −30% +2% +15% +60% −5% −40% +90% −80% +30% −30% +5% +8% −40% +5% +5% −50% −10% +5% −50% +15% −12% −25% +10% −15% −25% −10% −15% −3% −11% −3% +43% +46% +46%	<33 nT >> >> >> >> >> >> >> >> >> >> >> >> >> >> >> >> >> >> >> >> >> >> >> >> >> >> >> >> >> >> >> >> >> >> >> >> >> >> >> >> >> >> >> >> >> >> >> >> >>	1 h 4 h 24 h 48 h 10 min 1 h 4 h 48 h 96 h 10 min 4 h 48 h 96 h 10 min 1 h 4 h 24 h 96 h 4 h 48 h 10 min 1 h 4 h 48 h 96 h 10 min 4 h 24 h 48 h 96 h 10 min 1 h 4 h 24 h 48 h 96 h 10 min 1 h 4 h 24 h 48 h 96 h 1 h 4 h 1 h 96 h 1 h 48 h 48 h 48 h	3 >> >> >> >> >> >> >> >> >> >> >> >> >> >> >> >> >> >> >> >> >> >> >> >> >> >> >> >> >> >> >> >> >> >> >> >> >> >> >> >> >> >> >> >> >> >> >> >> >>	Paired Student’s *t*-test, and Bonferroni post hoc test, and Hochberg (BH) multiple testing correction	Magnetometer, 3-axis, spatial distribution, GMF: 41.94 μT	Helmholtz coils (3-axis)	Ø 128 cm	0.41 (Q2)	[92]
15	*Arabidopsis thaliana* Columbia ecotype Col-4, seedlings	cry2 phosphorylation rate, cry2 dephosphorylation rate	−20% −15% −10% −20% −20% −10%	<50 nT >> >> >> >> >>	30 60 90 30 60 90 min	3 >> >> >> >> >>	Student’s *t*-test	Magnetometer, 3-axis, spatial distribution, GMF: ~45 μT	Helmholtz coils (axis)	Ø 88 cm	0.6 (Q2)	[102]
16	*Arabidopsis thaliana*, seedlings, wild type or *cry1cry2* mutants, *phyAB* mutants	Seed germination: Wt Blue light: 50, 60, 70 h Darkness: 50 h *cry1cry2* mutants Blue light: 50, 60, 70 h, darkness Hypocotyl length Wt Blue light Darkness *cry1cry2* mutants Blue light Darkness *phyAB* mutants Blue light, Darkness	−50% −60% −45% −50% −80% −50% −40% N/A N/A −50% −30% −40% −40% N/A	<200 nT >> >> >> >> >> >> >> >> >> >> >> >> >>	96 h >> >> >> >> >> >> >> >> >> >> >> >> >>	50 >> >> >> >> >> >> >> >> >> >> >> >> >>	Student’s *t*-test	Magnetometer, 1-axis, spatial distribution, GMF: ~50 μT	μ-Metal chamber and Helmholtz coils (1-axis)	25 cm × 40 cm Ø 18 cm	0.68 (Q1)	[103]

N/A—effect was not observed,. ANOVA—analysis of variance, spatial distribution—the authors indicate an assessment of the spatial distribution of the magnetic field; numerical values of variation are given, 3D map—a detailed image of the spatial distribution of magnetic field induction is given.

### 3.3. Effects of HMC on Cell Level

**Figure 5:**
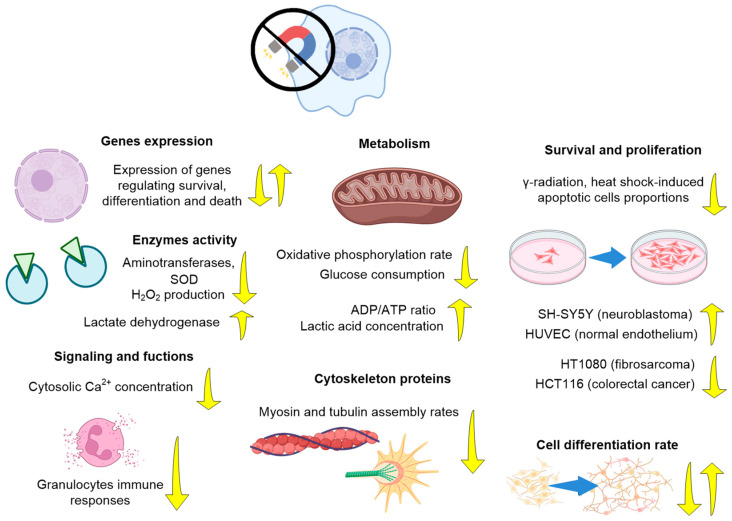
Main directions of effects of hypomagnetic conditions at the molecular–cellular level. Arrows indicate the direction of the effect: decrease (down) or increase (up) of the parameter (references in Table 1).

The effects of HMC on the cellular level are highly dependent on the cell types [104] (Figure 5, Table 3). For a primary culture of neurons in the brain of newborn mice, HMCs increase the proliferation rate [30]. For SH-SY5Y neuroblastoma cells (field induction higher than in the previous study), a decrease in the proliferation rate and an extension of the S phase of the cell cycle were found [47]. The effects of HMC depend in a complex way on the residual magnetic field induction [105]. Using the SH-SY5Y line as an example, it was shown that during HMC, there was expression of over 2400 genes involved in the regulation of cell survival and death, with 90% of genes experiencing a decrease in expression, and only 10% an increase [49].

Using a primary culture of the mouse hippocampus, it was shown that HMCs increase the cell size and proliferation rate, but reduce the expression of Nestin, Neurod1, GFAP, and βIII-tubuline proteins [30]. An increase in the proportion of noradrenergic neurons, GABA, and taurine concentrations in the brain was also found [59,106]. In other studies, under the influence of HMC, a decrease in the expression of maturity markers in embryonic stem cells during neuronal differentiation was observed [107]. Similar results were obtained during the differentiation of mouse hippocampal neurons in vivo [60]. At the same time, morphological (shortening of dendrites) and molecular changes (decreased expression of cell maturity markers) of neurons were observed. The decrease in the proportion of differentiated cells was caused by the triggering of signaling cascades that negatively regulate differentiation by oxidative stress [60]. The described changes at the cellular level led to the changes in behavioral reactions described above. It is expected that changes in the brain at the cellular level should also change its physiological state.

All of the above is consistent with the fact that HMCs contribute to the disruption of oxidative phosphorylation in mitochondria. In addition, disruption of myosin packaging and increased death of femoral muscle myocytes were recorded in the HMC [86].

Other studies found that with HMC (12 nT, 2 days), cell proliferation increases, while the duration of mitosis decreases [108]. Several studies with primary cultures of normal endothelial cells (HUVEC) did not find an effect of HMC (~300 nT, 7 days) on proliferation [109]. One possible mechanism for HMC’s impact on bone tissue (see above) could be alterations in iron metabolism, as well as a diminished rate of osteoblast differentiation and heightened activity of osteoclasts [70]. Another possible mechanism for HMC-related impairments in osteoblast differentiation could be changes to both iron and calcium metabolism [110]. Furthermore, alterations in the cell cycle (reduction in S phase, elongation of G2/M phase) and variations in the expression of osteoblast matrix protein regulators and mineralization rate are noticeable in HMC [110].

There is evidence of the potential carcinogenic effect of weak magnetic fields [111]. Hypomagnetic conditions (~2 μT) reduce oxidative stress, namely, H_2_O_2_ production, in the fibrosarcoma HT1080 and pancreatic cancer AsPC-1 cell lines [112] and protect the leukemic cell lines HL-60, HL-60R, and Raji from apoptosis, caused by heating [113]. However, in other work, HMC (5 days, 500 nT) caused an increase in lipid peroxidation [114,115]. Notably, mutations in the retinoid receptor (HL-60R lineage) did not affect the effects of HMC [113]. The effects of the HMC on cell division in a culture may depend on the concentration of FBS (fetal bovine serum) in the culture medium; the higher the serum concentration, the higher the effect of the magnetic field [108]. One of the mechanisms for triggering signaling cascades in cells by HMC is a decrease in the concentration of Ca^2+^ in the cytoplasm of cells [109].

The expression of cryptochrome genes *cry1*, *cry2*, and *AKH* (adipokinetic hormone) and *AKHR* (adipokinetic hormone receptor) was altered in the HMC, implying a role for cryptochrome and adipokinetic hormone-dependent signaling in modulating insects’ phototaxis and behavior. The observed effects were significantly sex-dependent in insects [72]. The variability in gene expression of *EF1-α*, *16S*, *ACT1*, *ARF1*, *RPS15*, *α-TUB1*, *AK*, and *RPL5* in insects was found to depend on the developmental stage (imago or nymphs), sex, and morphology (macropterous or brachypterous) after the application of HMC [116]. It should be noted that the methods of assessing the stability of genetic expression (BestKeeper, NormFinder, GeNorm, and comprehensive analyses) provide additional information on the effects of HMC at the molecular level, including when no significant effects were detected with a classical cycle threshold (Ct) analysis [116].

### 3.4. Effects of HMC at the Molecular Level In Vivo

HMCs inhibit the activity of superoxide dismutase (as a consequence, a decrease in H_2_O_2_ production) [39]. HMC led to a decrease in O_2_ consumption by cells, but the expression of respiratory chain proteins *blw* (the catalytic subunit F1 ATP synthase) and cytochrome c1 did not change [51]. There is also evidence of a decrease in the ATP/ADP ratio and mitochondrial potential under hypomagnetic conditions. At the same time, an increase in glucose consumption and lactate concentration and an increase in lactate dehydrogenase activity occur in the cells [50]. With HMC, the rate of human tubulin assembly in vitro decreases almost two-fold. It is noteworthy that after the addition of tau protein, tubulin assembly is almost completely inhibited [35]. Actin assembly in neuroblastoma cells is also inhibited in the HMC [47]. Deterioration of actin assembly contributes to a decrease in migration and adhesion of neuroblastoma cells, and changes in morphology [47]. Nitrogen transport in the body also changes under the influence of HMC: a decrease in the activities of blood aspartate and alanine transferases is observed [81]. Interestingly, hypomagnetic conditions may have a radioprotector effect. Using normal human fibroblasts, it was shown that HMCs reduce the number of DNA fragmentations both in the control and after irradiation with 0.5 Gy of γ-radiation [117]. HMCs also affect chromatin condensation in human cells. HMCs contribute to the accumulation of heavy metals Fe, Mn, Co, Ni, Cr, and Cu in the cells (muscles, brain, etc.) [29,67].

**Table 3:** Examples of the effects of HMC cellular and molecular levels (animals).

№	Biological Object	Characteristics	Effect, %	Magnetic Flux Density	Time	N	Statistic	Validation	Experimental Setup	Size or Volume	SJR	Ref.
1	Mice *(M. musculus)* C57BL/6 J adults, 8–10 weeks	Proportions of hippocampal neuron types: BrdU+ cells BrdU+ Grap+ SOD2+ type1 cells BrdU+ Grap+ SOD2+ type1 cells Expression of negative regulation of proliferation genes Expression of oxidative stress response genes	−12% −12% −25% +10^2^–10^5^ times −10^2^–10^5^ times −8%	170 nT >> >> >> >> >>	8 weeks >> >> >> >> >>	10 >> >> >> >> >>	One-way or two-way ANOVA or Student’s *t*-test	Magnetometer, 3-axis, spatial distribution, ambient magnetic fields, noise, and light were measured. SMF in incubator: 39.4 ± 3.6 μT. AMF: 50 Hz Bt PSD1/2 2.37 nT/√Hz	Helmholtz coils (3-axis)	Ø 50 cm	5.12 (Q1)	[60]
2	Mice *M. musculus* line C57BL/6 J newborns	Proportions of hippocampal neuron types: BrdU+ cells BrdU+ GFAP+ S100β- cells BrdU+ Ki67+ DCX- cells BrdU+ Ki67+ DCX- cells BrdU+ DCX+ NeuN+ cells BrdU+ DCX- NeuN+ cells Dendrite length	−15–25 −50% −60–99% −60–80% −5–30% −40–50% −5%	0.17 μT >> >> >> >> >> >>	4 weeks >> >> >> >> >> >>	6 >> >> >> >> >> >>	One-way or two-way ANOVA or Student’s *t*-test	Magnetometer, 3-axis, spatial distribution, ambient magnetic fields, noise, and light were measured. SMF in incubator: 39.4 ± 3.6 μT. AMF: 50 Hz Bt PSD1/2 2.37 nT/√Hz	Helmholtz coils (3-axis)	Ø 50 cm	5.12 (Q1)	[60]
3	Mice, *Mus musculus* line C57BL/6 neonatal, young (P15), adult (2 months)	Primary brain culture from a region of the brain, hippocampus: Cell diameter, proliferation rate The expression of proteins Nestin, Sox2, Neurod1, GFAP, βIII-tubuline	+50% +30% −50%	<85 nT >> >>	7 days >> >>	24 >>	One-way ANOVA and χ^2^ test	Magnetometer, spatial distribution Local MF for cells (incubator): 15.1 ± 2.2 μT GMF for animals: 49.88 ± 1.82 μT	Magnetic shielding chamber and Helmholtz coils (3-axis)	Ø 40 cm	3.37 (Q1)	[30]
4	Human neuroblastoma cell line SH-SY5Y	H_2_O_2_ production Superoxide dismutase activity Cell cycle phase ratio: proportion of S phase in the cell cycle	−50% −60% +200%	<500 nT >> >>	16 h >> >>	3 >> >>	Shapiro–Wilk test, one-way ANOVA, Bonferroni post hoc test	Magnetometer 3-axis 3D map GMF: ~45 μT	Permalloy chamber	10 cm × 10 cm × 10 cm	3.37 (Q1)	[39]
5	Human neuroblastoma cell line SH-SY5Y	Expression of genes regulating survival, cell division, adhesion, apoptosis, functions (a total of 2464 analyzed)	+216 genes −2248 genes	<200 nT >>	1–4 days >>	6 >>	One-way ANOVA	Magnetometer, 3-axis, spatial distribution, AFM (control): 50 Hz, 575.7 ± 29.1 nT AFM (experiment): 50 Hz, <12.0 nT	Permalloy chamber	0.24 m^3^	1.45 (Q1)	[49]
6	Ansell’s mole-rats (*F. anselli*), adult	Number of c-Fos-IR+ cells Subcortical nuclei, cortical regions, hippocampus, striatum, and primary motor and primary somatosensory cortices	−50% −40% +60%	~300 nT >> >>	1 h >> >>	22 >> >>	One-way ANOVA, Tukey post hoc test	Magnetometer, 3-axis, spatial distribution, HMF variation: <1% GMF: ~46 μT	Helmholtz coils (1-axis) and μ-metal chamber	Ø 170 cm 2 m × 2 m × 2 m	1.2 (Q1)	[78]
7	Mice, C57BL/6J, 7 weeks old	ROS levels in hippocampus: DG region, CA region Gene expressions: NADPH oxidase 4, eosinophil peroxidase, keratin 1, nitric oxide synthase 2, glutathione peroxidase 3, heat shock protein 1A	+30% +30% +155% +85% +86% +60% −70% −64%	31.9 nT >> >> >> >> >> >> >> >>	8 weeks >> >> >> >> >> >> >> >>	4 >> >> >> >> >> >> >> >>	Double-blind study, unpaired Student’s *t*-test	Magnetometer 3-axis 1 point, time distribution, HMF variation: < 4.5 nT GMF: ~55 μT Temperature, illumination, and relative humidity equal in all conditions	Helmholtz coils (3-axis)	2 m × 2 m × 2 m	1.15 (Q1)	[61]
8	*Drosophila melanogaster* sperm	Cell mobility Oxygen consumption by cells (pmolO_2_/_mL_/min/test) Protein expressions: *blw* (the catalytic subunit F1 ATP synthase), c1 cytochrome, cyt c1 oxidase	−30% −25% N/A	<1 nT >> >>	6 h >> >>	200 >> >>	One-way ANOVA, Student’s *t*-test	Magnetometer, 1-axis, 1 point, GMF: 48 μT	Helmholtz coils	-	1.15 (Q1)	[51]
9	Black Garden Ant (*Lasius niger*)	Gene expression: *MagR* *cry* Protein content: SOD GSR H_2_O_2_ content Endogenous amine concentrations: tyramine (TA), octopamine (OA), L-DOPA, dopamine (DA), serotonin (Ser), melatonin (Mel)	+20% −18% +38% −20% −60% −20% −80% −80% −75% −80% +10%	~40 nT >> >> >> >> >> >> >> >> >> >>	14 days >> >> >> >> >> >> >> >> >> >>	30 >> >> >> >> >> >> >> >> >> >>	Kolmogorov–Smirnov test, one-way ANOVA, Tukey’s post hoc test	Magnetometer, 3-axis, spatial distribution, HMF variation: < 6 µT GMF: ~42 µT GMF variation: < 20 nT	Helmholtz coils (3-axis)	Ø 128 cm	1.15 (Q1)	[66]
10	Tardigrades *(Paramacrobiotus experimentalis)* females and males of different age	Mitochondrial potential	−6%	<250 nT	15 days	45	Two-way ANOVA, Tukey post hoc test	Magnetometer 1-axis 1 point GMF: ~50 μT	μ-Metal shielding chamber (approximately 77% nickel, 16% iron, 5% copper, and 2% molybdenum)	18.5 cm × 12 cm × 33 cm	1.03 (Q1)	[76]
11	Human neuroblastoma SH-SY5Y	Migration and adhesion (rate, distance, cell count) Morphology (outgrowth width) Actin assembly in vitro	−40% - −50% −10%	<200 nT >> <500 nT	4 days >> 48 h	4 >> 6	One-way ANOVA, Chi-square test, Kolmogorov–Smirnov test	Magnetometer, 3-axis, spatial distribution AMF: 12.0 ± 0.0 nT at 50 Hz (in permalloy chamber) SMF: 15.1 ± 2.2 μT; AMF: 575.7 ± 29.1 nT at 50 Hz (incubator) SMF: 52.5 ± 0.4 μT; AMF: 14.0 ± 1.0 nT at 50 Hz (control animals)	Permalloy chamber Helmholtz coils (3-axis)	50 cm × 50 cm × 50 cm Ø 40 cm	0.97 (Q1)	[47]
12	Mouse embryonic stem cells (mESCs) differentiate into neuronal cells	Expression of neuronal differentiation markers: Huj1 Map2 Proportion of differentiated cells Brachyury expression	−90% −75% −80% −80%	<10 nT >> >> >>	12 days >> >> >>	3 >> >> >>	Shapiro–Wilk test, one-way ANOVA, Bonferroni post hoc test, Student’s *t*-test (normal distribution)	Magnetometer, 3-axis, 1 point	Helmholtz coils (3-axis)	-	0.97 (Q1)	[107]
13	Oriental armyworm; *Mythimna separata* eggs, larvae, pupae, and imago (females and males)	Vitellogenin *Vg* gene expression	−50%	<500 nT	12 h	300	One-way or two-way ANOVA	Magnetometer, 1-axis, 1 point, time distribution, HMF variation: < 500 nT	Helmholtz coils	Ø 50 mm	0.94 (Q1)	[73]
14	Human neuroblastoma cell line SH-SY5Y	Number of cells in a culture Proliferation rate Number of cells in G0 phase Number of cells in G1 phase Number of cells in G2/M phase	+8% +8% +7% −7% −5%	<150 nT >> >> >> >>	2 days >> >> >> >>	3 >> >> >> >>	One-way ANOVA	Magnetometer, 3-axis, spatial distribution, temperature and relative humidity equal in all conditions, GMF (incubator): < 11 μT GMF (laboratory): ~56 μT	Permalloy chamber	0.24 m^3^	0.89 (Q1)	[108]
15	Fibrosarcoma HT1080 and pancreatic AsPC-1 cancer cells	H_2_O_2_ production	−12%	500 nT	24 h	3	One-way ANOVA	Magnetometer, 3-axis, spatial distribution, HMF variation: 0.5–2 µT Temperature variation: < 0.1 °C GMF: ~45 μT	μ-Metal cylinder and Helmholtz coils (3-axis)	Ø 12.5 cm	0.89 (Q1)	[112]
16	Cow (*Bos taurus*) and human (*Homo sapiens*)	Self-assembly rate of tubulin from α/β-subunits: no tau protein in the presence of tau (recombinant human tau23) protein	−40% −90%	10–100 nT >>	20 min >>	7 >>	Tsou’s method	Magnetometer 1-axis 1 point GMF: ~50 μT	Helmholtz coils (1-axis)	Ø 40 cm	0.79 (Q1)	[35]
17	Human neuroblastoma cell line SH- SY5Y	Proliferation rate Glucose consumption Lactic acid concentration Lactate dehydrogenase activity ATP concentration ADP/ATP ratio Mitochondrial potential	+12% +22% +18% +7% +13% −9% −10%	<200 nT; >> >> >> >> >> >>	72 h >> >> >> >> >> >>	3 >> >> >> >> >> >>	Two-way ANOVA, Tukey’s post hoc test (multiple comparisons, Student’s two-tailed *t*-test (two groups)	Magnetometer, 3-axis, spatial distribution, AMF: 50 Hz, <12.0 nT SMF (control incubator) 15.1 ± 2.2 μT; AMF: 50 Hz (control incubator), 575.7 ± 29.1 nT	Permalloy chamber	50 cm × 50 cm × 50 cm	0.79 (Q1)	[50]
18	Brown planthopper, *S. furcifera* males and females, imago	Gene expression cry1 cry2 Adipokinetic hormone concentration Expression Adipokinetic hormone receptor	−20% +10% +10% −17% +25%	~477 nT >> >> >> >>	1–5 days >> >> >> >> >>	40 >> >> >> >> >>	One-way or two-way ANOVA	Magnetometer, 1-axis, spatial distribution (0–1.06 μT) GMF: ~50 μT	Helmholtz coils (3-axis)	Ø 30 cm	0.74 (Q1)	[45]
19	Human bronchial epithelial cell line BEAS-2B after X-ray exposition (1 Gy/min)	Survival, DNA fragmentation, γH2AX expression, Colocalization coefficient of γH2AX and p53BP1	+6% 0% −40% +40%	50 nT. >> >> >>	30–320 min >> >> >>	3 >> >> >>	One-way ANOVA	Magnetometer, 1-axis, spatial distribution, SMF (incubator): 6–13 μT GMF: ~47 μT	Permalloy chamber Helmholtz coils (3-axis)	Ø 40 cm	0.43 (Q3)	[118]
20	Human fibrosarcoma cell line HT1080 and human colorectal cancer cell line HCT116	Proliferation	−19%	200 nT	1–3 days	9	One-way ANOVA	Magnetometer, 1-axis, spatial distribution, SMF (incubator): 6–13 μT GMF: ~43 μT	Helmholtz coils (3-axis)	Ø 50 cm	0.43 (Q3)	[104]
21	Jurkat cells	Anti-CD3-antibody-induced Ca^2+^ influx characteristics: Basal slope: G0/G1 phase cells, S phase cells Reak: G0/G1 phase cells, G2-M phase cells Active intercept: G0/G1 phase cells, S phase cells, G2-M phase cells Active average: G0/G1 phase cells, G2-M phase cells	+20% −10% +4% −12% +104% +83% +81% +82% +65%	<300 nT >> >> >> >> >> >> >> >>	20 min >> >> >> >> >> >> >> >>	10 >> >> >> >> >> >> >> >>	MANOVA or paired Student’s *t*-test	Magnetometer, 1-axis, 1 point AMF variation: <1 nT	μ-Metal chamber	33 cm × 38 cm × 20 cm	0.43 (Q3)	[119]
22	Human umbilical vein endothelial cells (HUVECs)	Proliferation eNOS expression VEGF gene expression	N/A N/A N/A	300–500 nT	24 h	3	Student’s *t*-test	Magnetometer, 3-axis, spatial distribution, SMF (incubator): 6–12 μT	Helmholtz coils and μ-metal chamber	8.5 cm × 12.5 cm × 6.5 cm	0.43 (Q3)	[34]
23	Mice *M. musculus* line C57BL/6 newborns (E18)	Viability of femoral muscle myocytes Proportion of cells in apoptosis and necrosis Myosin packaging quality Residual glucose, mM Glycogen, μmool/g protein ATP, μmool/g protein ADP/ATP ratio	−5–10 N/A qualitatively +10% +10% −60 +60–80	<1 μT >> >> >> >> >> >>	3 days >> >> >> >> >> >>	11 6 12 >>	One-way ANOVA or Student’s *t*-test	Magnetometer, 3-axis, 3D map SMF (control incubator): 38–55 μT AMF: 55–62 Hz, 105. ± 19.2 nT	Helmholtz coils (3-axis)	Ø 40 cm	0.42 (Q3)	[86]
24	Human adults, healthy blood cells	Activity of aspartate aminotransferase Activity of alanine aminotransferase Hemolysis	−12% −28% +9.5 times	100 nT >> >>	72 h >> >>	10 >> >>	Student’s *t*-test	Magnetometer, 1-axis, 1 point GMF: ~50 μT	Helmholtz coils	-	0.4 (Q3)	[81]
25	Mice *M. musculus* line CD-1 adults 24–26 g, males	fMLF or PMA induces ROS production by peritoneal granulocytes	−25%	20 nT	1.5 h	10	Student’s *t*-test	Magnetometer, 1-axis, spatial distribution Ambient GMF: ~42 μT AMF: 50 Hz, 15-50 nT	Permalloy chamber	-	0.18 (Q4)	[82]
26	Rat (*Rattus norvegicus*) newborns	Cytosolic Ca^2+^ concentration	−8%	~300 nT	7 days	3	Student’s *t*-test	Magnetometer, 1-axis, 1 point GMF: ~48 μT	Nanomaterial-based ASM AMAG 172 chamber	-	0.18 (Q4)	[109]
27	Mice *M. musculus* C57BL/6 (4–6 weeks old), male	Condition of skeletal muscle cells Citric acid concentration in muscles Number of SS mitochondria Mitochondrial length	qualitatively −30% −20% +15%	1.12 μT >> >> >>	30 days >> >> >>	10 >> >> >>	Kolmogorov–Smirnov test, one-way ANOVA, Student’s *t*-test, or Mann–Whitney U-test	Magnetometer 3-axis 3D map SMF variation: < 430 nT AMF: 120 Hz, <230 nT	Helmholtz coil (3-axis)	Ø 40 cm	0.13 (Q4)	[85]
28	Rat, *Rattus norvegicus* line Wistar	Proportion of c-fos+ neurons in the thalamus Proportion of active MOROP3+ neurons in the thalamus and periaqueductal area Proportion of active MOROP3+ neurons in the frontal cortex and superior colliculus	−20% −80% −2%	50–150 nT >> >>	21 days >> >>	12 >> >>	Wilcoxon signed-rank test, Kolmogorov–Smirnov test	Magnetometer, 3-axis, 1 point, HMF variation: < 50 nT	Helmholtz coils	Ø 50 cm	-	[58]
29	Brown planthopper *Nilaparvata lugens* adults, macropterous and brachyppterous	Stability of expression of *AK* and *α-Tub1*	−75%	523 nT	2000 h		One-way ANOVA, benchmarks of Cohen for small effects	Magnetometer, 3-axis, one point, HMF variation: < 2% GMF: 50 μT	Helmholtz coils	Ø 120 cm	1.03 (Q1)	[116]
30	Murine osteoblastic cell line MC3T3-E1	Cell proliferation, Cell area Cell cycle phase duration: S, G2/M, Fe concentration in medium, Ca concentration, Nodule area, Total protein Gene expression: *ALP*, *BSP*, *CoI*, *DMP1*, *OC*, *TfR1*	N/A +20% −20% +20% −10% −20% −60% +10% +20% −15% +40% −30% +80% +80%	500 nT >> >> >> >> >> >> >> >> >> >> >> >> >>	48 h >> 24 h >> 8 days 8 days >> >> 8 days >> >> >> >> >>	3 >> >> >> >> >> >> >> >> >> >> >> >> >>	One-way ANOVA, Newman–Keuls test	Magnetometer, spatial distribution, AMF in control incubator 50 Hz ~1 μT AMF in an experimental incubator 50 Hz, < 12 nT GMF: ~45 μT	Permalloy chamber	550 × 420 × 420 m	0.73 (Q1)	[110]

N/A—effect was not observed, ANOVA—analysis of variance, spatial distribution—the authors indicate an assessment of the spatial distribution of the magnetic field; numerical values of variation are given, 3D map—a detailed image of the spatial distribution of magnetic field induction is given.

### 3.5. Effects of HMC on Bacteria

The effects of hypomagnetic conditions on bacteria have been studied in relative detail in two aspects. The first is the functioning of magnetosomes in magnetobacteria. The second is the possible impact on antibiotic resistance of bacteria (Table 4). For magnetotactic bacteria, it has been shown that hypomagnetic fields do not affect the number of magnetosomes, but reduce their size, differentially change gene expression, and disrupt the ability of bacteria to migrate in the thickness of liquid [32,120]. A hypomagnetic field can reduce bacterial resistance to antibiotics, but the effect is highly dependent on the strain and antibiotic used. HMCs may not affect and sometimes even increase the minimum inhibitory concentration (MIC) against a particular antibiotic [121,122]. An analysis of bacteria in the nasopharynx of astronauts on board the International Space Station (ISS) and a mathematical model based on these experimental data made it possible to determine that HMCs can cause a significant decrease in antibiotic resistance of bacteria [123]. According to measurements, the magnetic field induction on board the ISS is ~15–40 μT [124,125].

### 3.6. Effects of HMC on Solutions

The effects of HMC and magnetic fields in general can also manifest themselves at the level of water and aqueous solutions. There is a lot of experimental data that recorded the effects of water treated in MP, which was then used for the growth of cell cultures, watering plants, or drinking for humans and animals [126,127,128,129]. Thus, in [130], a combined MF with a constant component of 60 μT and a variable component of 100 nT affected neutrophil suspensions, and indirectly through aqueous solutions.

Such effects are often dependent on impurities in the water. In particular, it was shown in [131] that a constant MF caused an increase in the concentration of hydrogen peroxide in a solution from nanomoles to micromoles, and the effects of constant and radio frequency MF depended on the presence of dissolved gases in water and disappeared when the initial solutions were degassed. In another study, the effects of HMF changed both with mechanical impact (shaking) and with changes in gas composition (purging with argon) [132]. It is known that the conditions for obtaining and storing water and aqueous solutions affect the physical parameters of solutions, probably due to changes in the concentration of dissolved gases and the appearance of nano-sized gas bubbles [133]. Exposure to magnetic fields, both fairly strong with an induction of ~300 mT and weak (~10 μT), also led to changes in the physical characteristics of aqueous solutions [134].

Among all physical parameters, when exposed to MFs, experimenters most often note changes in a dielectric constant [135,136]. It was also found that variations in the dielectric constant of water depend on external conditions: mechanical disturbances such as stirring and pouring and relaxation to the initial state for more than an hour [137].

Another parameter that is often used to record the effects of MFs is the luminescence of water and aqueous solutions of proteins [138,139]. Altered magnetic fields (3.7 Hz frequency and induction of 0.04 μT, induction of constant MF, 42 μT) increased the luminescence intensity several times during 2–4 h of treatment [139]. Stirring the treated water had a specific effect on the protein, similar to what happened when the protein solution was directly treated with a magnetic field. The magnetic field does not affect the chemiluminescence parameters of water but changes the intensity and standard deviation of the chemiluminescence intensity of aqueous solutions of IgG [138]. The severity of the effect depends both on the frequency of the applied magnetic field and on the protein concentration.

**Table 4:** Examples of the effects of HMC on bacteria.

№	Biological Object	Characteristics	Effect, %	Magnetic Flux Density	Time	N	Statistic	Validation	Experimental Setup	Size or Volume	SJR	Ref.
1	*Pseudomonas* (strain P3) *Enterobacter* (strain E1)	MIC for antibiotics: ampicillin, kanamy, tetracycline, ofloxacin, ceftazidime, tetracycline, ofloxacin	+80% −90% +30% −30% −50% −80% −60%	<500 nT >> >> >> >> >> >> >>	6 days >> >> >> >> >> >> >>	6 >> >> >> >> >> >> >>	Student’s *t*-test	Magnetometer, 1-axis, 1 point	Helmholtz coils	-	0.55 (Q2)	[122]
2	*Magnetospirillum magneticum*	Magnetosome size Gene expression: *mms13*, *mms6*, *magA*	−9% +70% −10% N/A	<500 nT >> >> >>	16 h >> >> >>	>300 >> >> >>	Two-way ANOVA, two-tailed Student’s *t*-test, Mann–Whitney U-test	Magnetometer, 3-axis, spatial distribution, stability HMF area: 200 mm × 200 mm × 200 mm	Helmholtz coil	Ø 1050 mm	0.53 (Q2)	[32]
3	*E. coli*	MIC for antibiotic (proportions of analyzed strains): ofloxacin, kanamycin, tetracycline, ceftazidime, ampicillin	−9% +12% −19% +12% −10%	~40 nT >> >> >> >>	6 days >> >> >> >>	6 >> >> >> >>	Two-tailed Student’s *t*-test	Magnetometer, 1-axis, 1 point	Helmholtz coils	Ø 40 cm	0.4 (Q3)	[121]
4	*Escherichia coli* strain K12 AB1157 in stationary growth phase	Maximum relative viscosity	−18% +18%	30, 60, or 80 nT 45, 70, or 95 nT	15 min >>	15 >>	Student’s *t*-test	Magnetometer, 3-axis, spatial distribution, AFM: 50 Hz, <30 nT	Helmholtz coils (2-axis)	Ø 19.6 cm	0.43 (Q3)	[140]

N/A—effect was not observed.

## 4. Potential Effects of HMC on Organisms Depending on Induction

In this section, we will try to give a brief description of the “symptoms” of being in the HMC. As mentioned earlier, three ranges of HMC induction can be distinguished, which will correspond to the nearest near-Earth space objects: Mars (300 nT–5 μT), the Moon (10–300 nT), and interplanetary space (0.1–10 nT). In this section, we will try to summarize the possible sets of problems that may arise in these ranges of HMC induction.

Under “Martian” magnetic conditions closer to Earth’s, the following effects can be expected: disturbances in the musculoskeletal system and glucose metabolism [86], as well as a decrease in central nervous system lability and changes in brain rhythms [41,62]. Even in such relatively mild HMCs, teratogenesis cannot be excluded [87].

In “Lunar conditions”, disorders of the central nervous system are also possible, but at a more complex level—a slowdown in the processes of neuronal differentiation and deterioration in cognitive functions, and an increase in aggression [30,58,63,78]. Impairments in the functioning of the musculoskeletal system, both muscles and bones, and tissue regeneration are also possible [35,69,85]. Changes in the functioning of the cardiovascular system are also possible with HMC of the Moon, but their long-term effect cannot yet be predicted unambiguously [80,141]. Changes in nitrogen transport and metabolism are possible in HMC [81]. It is noteworthy that for these HMUs, an increase in DNA repair was noted in response to heat shock and ionizing radiation [113,117]. With these HMCs, changes begin to appear at the molecular and cellular levels; in particular, carcinogenesis increases and tubulin self-assembly is impaired [30,34,35,50].

The risks associated with interstellar flights largely coincide with “Lunar conditions” (Table 1). Additional consequences may include changes in the metabolism of metals with their subsequent accumulation in tissues [29,67]. Perhaps the peculiarities of metal absorption should be taken into account when planning the diets of astronauts. Suppression of the innate immune system should also be expected [82]. Changes in the cardiovascular system are also possible, and changes may affect the architecture of the heart tissue itself [44,84]. On the part of the central nervous system, architectural changes may also occur caused by a slowdown in neuronal differentiation [107].

Potential agriculture in “Martian” and “Lunar” conditions is expected to be different from Earth, even with the same lighting and temperature regimes. First, HMCs slow down the reproduction and development of insect pests of crops and their foraging behavior [45,64,68]. On the one hand, HMC will alter plant production, potentially leading to an increase in below-ground biomass and a decrease in above-ground biomass. On the other hand, a reduction in insecticide costs in HMCs may lead to some increase in plant production [96,100,101]. The time of germination, flowering, and fruiting will be elongated [88,90]. These factors should be considered when choosing crops for long-term stays in space.

Given the above, future space missions will be impossible without technology for protection from cosmic radiation, including the creation of artificial magnetic fields. Such technologies have been actively developed and partially implemented since the 1960s [142]. The main purpose is to protect equipment and crew from the destructive effects of cosmic radiation [143,144]. Currently, an active search for effective and cost-effective technology for protecting a spacecraft continues [145,146]. Protection can be divided into passive (shielding materials) and active (generation of magnetic field around the ship due to its power systems) [147]. Given the above, the strategy of creating an artificial magnetic field will be more attractive. It will provide, on the one hand, protection of the crew from cosmic radiation and, on the other hand, the “physiological level” of the induction of the surrounding magnetic field. The literature discusses ambitious projects to create artificial magnetic fields, including on a planetary scale for the colonization of Mars [148]. Several technological approaches have already been proposed, which the reader can familiarize themselves with in [148]; however, to select the most adequate and promising one, a sufficient amount of preliminary data is required.

## 5. Mechanisms of Action of Hypomagnetic Conditions on Living Systems

It has now been reliably established that magnetic field orders of magnitude lower than geomagnetic fields can cause biological effects (Table 1). The biological response to HMC can be viewed from a position that the geomagnetic field is an essential condition for the normal functioning of living systems at the level of chemical reactions; then, HMC can be considered as “a deprivation of normal physiological needs,” similar to the deprivation of other conditions: sleep, food, etc.

The general features of the effects of HMC on living organisms are as follows:

(1) The biological effects of magnetic fields often begin to be realized at low inductions (<1–2 µT), when possible thermal effects are excluded (Table 1). This leads to the so-called “*kT* problem” expressed by the inequality *mH* ≪ *kT*, where *H* is the magnitude of the magnetic field induction, *m* is the magnetic moment of the proposed magnet, *k* is the Boltzmann constant, and *T* is the effective temperature of the target [149]. The magnetic energy of an electron in a geomagnetic field is 2.9 × 10^–9^ eV, which is seven orders of magnitude less than *kT* at physiological temperatures. Energy values in hypomagnetic conditions are even lower. Thus, the energy approach to explain magnetobiological effects of this kind is meaningless [150].

(2) Biological effects have “amplitude windows” [37,81,105]. This is especially clearly seen in studies on bacteria and planarians, when an increase in induction causes alternately loss and restoration of the effect [140,151].

(3) The effects of HMC are poorly reproduced. In our opinion, the difficulty of reproducing biological effects is caused by many reasons: differences in non-magnetic conditions (temperature, humidity, etc.), external magnetic environment (presence of nearby electrical appliances, power lines, geomagnetic conditions, etc.), presence of an external electric field, internal heterogeneity of the simulated magnetic field, effects at the micro level, species of the organism, organ or cell line under study [34,39,152,153,154,155,156].

On the other hand, there is a fundamental cause of nonreproducibility, i.e., the randomness of manifestations of primary acts of interaction between targets and MF. This randomness can be circumvented at the expense of amplification. Here, we pass to the area of specific magnetoreception as in birds due to the radical pair mechanism (RPM) or in bacteria due to magnetosomes assembled from magnetite.

(4) This is probably why, apart from rare exceptions [35], there are no works on the effects on biomolecules in vitro. Therefore, the magnitude of the effect varies greatly at different levels of biological organization, even within the same organism: for example, between biochemical markers and behavioral responses.

This is especially clearly seen in works that combine several methodological approaches. For example, gene expression in HMC changes on a logarithmic scale, expression of differentiation markers changes linearly (sometimes four times), and a behavior changes linearly with less variability (no more than 20%) [60].

Today, magnetobiology distinguishes between specific (associated with special magnetoreceptors) and nonspecific magnetic effects [157]. The specific effects occur due to special magnetic receptors created by nature to help some animals survive, for example, during long seasonal migration routes. However, considerable interest today is also associated with nonspecific magnetic effects. At the end of the last century, it was believed that the magnetic field acts on a person bypassing the sense organs [158], that is, bypassing specialized receptors. However, considerable interest today is associated with nonspecific magnetic effects, since most of the magnetobiological effects are recorded in cells where there are no cryptochromes and magnetosomes [159]. Nonspecific effects are observed in many organisms: from protozoa and fungi to insects, plants, fish, animals, and humans [48,68,117,160]. Interest in these effects is growing because they can change many properties. In particular, gene expression changes [71,101,161,162]. In other words, magnetic conditions are one of the factors controlling and modulating protein synthesis. However, it has not yet been possible to use the capabilities of this gene control method, since the nature of the primary nonspecific magnetic field target in the body has not yet been clarified.

### 5.1. Probable Mechanisms of Static Magnetic Field Effects

**Figure 6:**
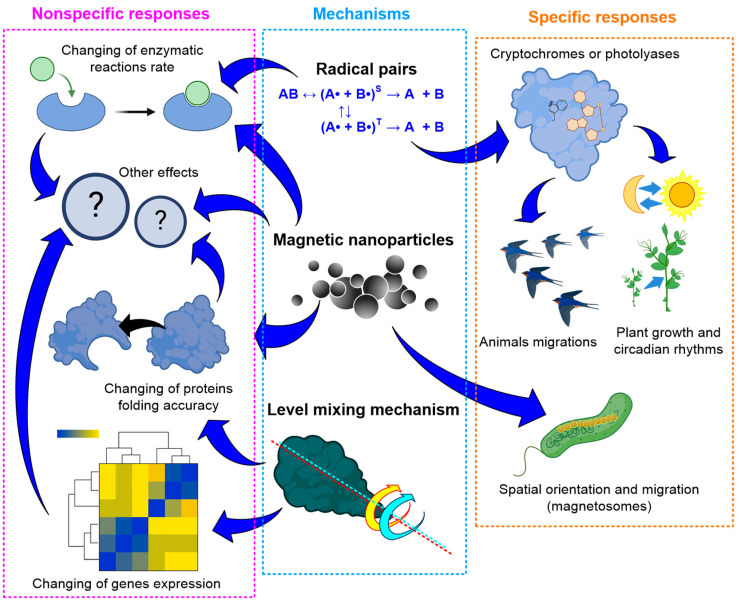
Proven or most probable mechanisms of action of magnetic fields on living systems of different levels, and their biological effects (references to the literature and explanations are in the text). Symbols "?" indicate currently unknown targets. Pink colour indicates non-specific effects of HMC. Orange colour indicates specific responses. Blue colour indicates mechanisms of action of magnetic fields on living organisms.

From 1980 to the present, many physical mechanisms and mathematical models have been proposed to explain the biological effects of weak magnetic fields. We will focus on those mechanisms of the biological action of magnetic fields that can most likely be realized in HMC or fields close to the Earth’s geomagnetic field (Figure 6). Mechanisms for alternating magnetic fields can be found in our other review [163]. In the literature, one can find several supposed mechanisms of the nonspecific action of magnetic fields on biological objects, which have different degrees of probability of actual manifestation in magnetobiology. The targets of magnetic fields can be molecules as a whole, protons, electron spins, and orbital magnetic moments [150,157,164,165,166]. The objects of these mechanisms are, respectively, single moments, a radical pair mechanism, and quantum rotations of molecular groups within proteins.

Among the main theoretical mechanisms of the action of static magnetic fields are the

(1) action of the Lorentz force on charged particles;
(2) participation of stable magnetic nanoparticles;
(3) radical pair mechanism;
(4) level mixing mechanism.

Unfortunately, at present, there is no 100% experimental confirmation of one or another mechanism. We will try to briefly characterize these mechanisms and their potential applicability in magnetobiology. The quantum mechanisms of these phenomena are described in detail in [167,168].

#### 5.1.1. The Action of the Lorentz Force on Charged Particles 

Another mechanism of the influence of magnetic fields is through the action on the movement of free ions due to the Lorentz force. Data with magnetic fields of high induction (1 T) and different directions showed that the direction of the magnetic field can affect the rate of synthesis of chiral molecules in the example of DNA, as well as the rate of proliferation of cell lines [169]. Many authors believe that the presence of the Lawrence force generated in the geomagnetic field is a key factor in the presence of chirality of biopolymers (DNA, proteins) at the dawn of life, which later became the cause of manifestations of asymmetry in living organisms. This assumption is confirmed by data on the possibility of excess synthesis of L-alanine in space (discovery of the amino acid on meteorites) and mathematical modeling of this process in conditions of polarized strong magnetic fields corresponding to conditions near nascent neutron stars [170,171,172]. However, in the case of a geomagnetic field, this effect is very small. According to calculations, the induction of a constant external magnetic field must be at least 10 times higher than the geomagnetic field for the effect of the Lorentz force to be observed in a living cell [167,168]. Therefore, it cannot be considered as an effector of biological effects in HMC magnetobiological studies [167].

#### 5.1.2. Nanoparticles with Magnetic Properties

Magnetite nanoparticles have been found in many organisms. In the geomagnetic field, their energy exceeds *kT*—the activation energy of chemical reactions [173,174]. Iron oxide nanoparticles must be attached to the cytoskeleton or to cells in the intercellular space to manifest magnetobiological effects. In this case, the energy of NPs (~100 nm in size) in a magnetic field is tens of times greater than the energy of thermal fluctuations (*kT*) [173], and the magnetic field sensitivity limit is approximately 200 nT [175]. On the other hand, the intrinsic magnetic field near such nanoparticles (~10–100 nm) reaches values of about 0.2 T [176].

Although the magnetosome mechanism seems to take place, it cannot explain all the observed magnetobiological effects. It is very difficult to explain the amplitude and frequency (for alternate MF) windows for magnetic nanoparticles’ biological effects via this mechanism since the natural frequencies of oscillations of a magnetic particle are much higher than the low-frequency range [156,177]. In addition, the effects of HMC can occur in organisms lacking magnetic nanoparticles. Therefore, the search for the molecular mechanism of magnetoreception continues. The simplest microscopic single-particle or multi-particle systems are often considered: a charged oscillator or a rotator with spin magnetic moments [178].

#### 5.1.3. Radical Pair Mechanism

There are several types of reactions in chemistry: combination, decomposition, replacement, and combustion reactions. Obviously, each of the reactions can be decomposed into initial molecule(s) and final molecules(s). Spin chemistry investigates reactions at the stage of intermediate products, where the reaction products create a short-lived pair, yet have not fused or decayed [166]. It is obvious that in the combination reaction at the physiological temperature, the energy reported by magnetic forces is many orders of magnitude less than the energy of diffusive motion; see the “*kT*” problem. For this reason, the weak magnetic fields cannot affect the process of convergence of initial reactants. It is the same for decomposition reactions; when the reaction products have dissociated, the magnetic field cannot affect the process of reverse recombination. The magnetic field can affect the reaction at this point [166,179].

As an example of a decomposition reaction, the following features of radical pair reactions can be emphasized:

AB ↔ (A• + B•)^T^ → A• + B•

↓↑

(A• + B•)^S^ → A• + B•

(1) The pair of radicals A• and B• are in a spin-correlated state. The transitions from the “T” to “S” state and vice versa are magnetosensitive. Direct reverse recombination from the “S” state to the original AB molecule is not possible. Therefore, conversion to the S state shifts product yields and reaction rates. Such reactions occur in enzyme–substrate complexes, so a magnetic field can modulate the release of free radicals. In the geomagnetic field, the energy of the S-T transition is orders of magnitude lower than the activation energy of a chemical reaction, so the magnetic field cannot be considered an initiator of the reaction but can be considered a modulator of its rate [180].

(2) The basis for the influence of a magnetic field on spin chemistry is the presence of a magnetic moment in a particle, collinear to its spin. Due to the complexity of the structure and composition of organic molecules, in addition to the spin moments of electrons, there are spin moments of protons and other magnetic nuclei, orbital motions of electrodes, and molecular groups with their charges.

(3) It is worth noting that the ST state of radical electrons differs from the ST state of whole molecules [167,176]. The electrons of the radicals are separated in space, and the exchange interaction is weak. In a molecule, a strong exchange interaction leads, according to the Pauli principle, to a significant energy gap between the S and T states, reaching about 1 eV for molecular oxygen. Therefore, magnetic effects for triplet and highly active singlet oxygen begin to be observed in fields of the order of 1 T, when the magnetic energy of the molecular electron is sufficiently high. For constant magnetic fields with an induction of 1–7 T, an increase in H_2_O_2_ generation due to the formation of singlet oxygen during the S-T transition has been experimentally shown [181,182].

(4) The mechanism of radical pairs has a low-frequency sensitivity due to the short lifetime of the correlated state of spins—10^–9^ s, rarely 10^–7^ s. This lifetime is the thermal relaxation time of the electron spins (unless the chemical process occurs too quickly). The relaxation time must be large enough for the magnetic field to noticeably change the state of the spins relative to each other. However, this is practically impossible to implement in biological systems at a temperature of ~300 K [156]. However, for permanent HMCs, this is not critical. The minimum magnetic field induction at which magnetic effects begin to occur can be calculated using the formula 1/γτ, where τ is the thermal relaxation time of the moment, and *γ* is the gyromagnetic ratio, or the ratio of the magnetic dipole moment of a particle to its angular momentum (depends on target) [164]. For electrons in enzyme–substrate complexes, τ = 10^–9^ s; in this case, the induction value is 5 mT, which is 100 times greater than the geomagnetic field. For an induction of at least 5 μT, often observed with nonspecific effects, the relaxation time must exceed 1 μs. It is still unclear whether conditions are possible in living tissue that ensure such a long relaxation with the participation of electrons [183]; however, in the case of a radical pair, a similar process is possible [184]. However, in bacteria and plants, the effects of magnetic fields have been found to depend on the reversal of the magnetic field and the frequency of the alternating magnetic field, and specific mechanisms such as radical pairs do not exhibit these properties [155]. The RPM is insensitive to magnetic field reversals because, in this case, the magnetic field changes the dynamics of a pair of magnetic moments relative to each other [67].

#### 5.1.4. Level Mixing Mechanism

There are theoretical difficulties in establishing the RPM mechanism. The first difficulty is the unreasonably long spin relaxation times of ~1 μs in fields of the order of GMF. The second difficulty is the magnitude of the biological effects in the GMF, which are much larger than theoretically expected [185]. In addition to these theoretical difficulties, there is a lot of experimental data that are difficult to explain in terms of RPM. ”

For example, bacteria do not express cryptochromes, but magnetobiological effects have been described for them [140]. The biological effects independent of a cryptochrome or the presence of illumination in the blue range were shown for *Arabidopsis thaliana* with deleted *cry1* and *cry2* genes. Some of the effects have been amplified by red light [94,100,103]. Such phenomena, as well as the effects of multiple maxima when changing the intensity of constant MF and the effects of magnetic field reversion, can be explained by the level mixing mechanism (LMM) [156,157]. LMM is based on the quantum mechanical principle that quantum levels of magnetic moments are mixed in a zero magnetic field. This effect is more general than in RPM. Therefore, it is possible to think of LMM as an extension of RPM.

The mechanism is based on non-uniform precession and thermal relaxation of the magnetic moment in the MF. In the LMM, a biological response is assumed to occur when the MF perturbs the dynamics of the magnetic moment during the relaxation time to such an extent that the deviation from the unperturbed uniform precession state becomes significant [156].

It is possible to assume that the primary sensors of weak magnetic fields in the model LMM may be molecules possessing magnetic spin and making rotational motions. These may be individual molecular groups in nucleic acids and proteins [156,177]. According to the available literature data, responses to HMC depend on the activity of gene expression, and, for example, are higher in seeds, where the processes of transcription and translation are more active [100]. Potential targets, in this case, are non-thermal rotations of RNA, DNA, enzymes, synthesized proteins, etc., accompanying gene expression. It can be assumed that the entire molecule carrying the magnetic sensor rotates [156]. According to the model proposed in work [156], the sensitivity of the sensor will depend on the rotation speed. This dependence is due to the overlap of two rotations: the precession of the magnetic moment of the sensor and the rotation of the sensor itself, which rotates along with its parent molecule. In the initial state, the axis of precession (magnetic field vector) and the axis of rotation of the molecule are located in the same way, for example, collinear, but the rotations themselves differ in speed. When a magnetic field is applied or removed, the rate of precession will change. If it becomes equal to the rotation speed, the magnetic moment vector will deviate relative to the sensor body. This fixed magnetic moment causes subsequent transduction of the magnetic signal to the level of biochemical reactions [140,186].

A theoretical justification for this mechanism was given in works [156,157,164]. With a gradual change in the magnetic field vector from parallel to antiparallel through zero, the response of the magnetic sensor, rotating with the parent molecule, shifts from zero. In this representation, responses of one biological system to magnetic fields of opposite directions but equal induction will differ. This phenomenon is not possible with the radical pair mechanism. Consequently, an analysis of the dependence of the quantitative biological effect on the induction of the applied magnetic field can provide information about the nature of the molecular processes of the nonspecific response of organisms to HMC [187].

According to calculation, the speed of movement of the selected angular position of the rotator does not depend on time and inertia-free action is possible, which solves the “*kT*” problem [156,188].

Molecular rotations are closely related to interference phenomena in molecules. Interference phenomena lead, for example, to the existence of atomic electron *p*-orbitals in the shape of a “dumbbell”. In the absence of valence interactions with the environment, the *p*-orbital rotates with angular velocity *γH*, where *γ* is the gyromagnetic ratio, or ratio of the magnetic dipole moment of a particle to its angular momentum [164]. In a hypomagnetic field, *H* → 0; therefore, the rotation of the orbital slows down, which increases the probability of a chemical reaction [178]. Quantum interference is a modern field of molecular electronics [189]. Interference effects in a magnetic field at the quantum level seem promising for research in the field of molecular electronics and theoretical magnetobiology [140,189]. A quantum mechanism in which a magnetic field changes the dynamics of a single moment relative to its surrounding molecular structure is attractive. It can explain, at least qualitatively, the features of nonspecific effects and offers numerical relationships for verification. The mechanism is as abstract and general as possible. Based on this mechanism, it is easy to construct a design of experiments to identify the nature of the biophysical sensor of nonspecific influences. There are few possible targets for a magnetic field—an electron, a proton, a magnetic nucleus, the orbital angular momentum of an electron, or a charged molecular group [164,187].

However, due to the novelty of the concept, LCM has yet to be experimentally validated compared to RPM, and its potential role in magnetoreception (including plants) remains largely unexplored.

### 5.2. Specific Responses

In the course of evolution, some animals have learned to utilize the primary acts of magnetic field interaction with targets, amplify them, and use them in navigation. Such organisms have developed a specific system of magnetoreception. The most striking example is the use of the mechanism of RPM in the cryptochrome signaling system in migrating animals and birds.

The presence of cryptochromes has been described both in animals (insects, vertebrates) and plants [94,190]. Cryptochromes can regulate gene expression in animals and plants, the concentration of phytohormones in plants, and behavioral responses of insects [31,49,90,94,95]. In the case of migrating birds, the localization of cryptochromes is assumed to be in double rods [150]. In the double rods of avian eyes, highly ordered structures of opsin dimers, oriented parallel within each cone, were found [191]. If we assume that cryptochromes are attached to such opsin dimers and are oriented in the same way, than they can synchronously respond to changes in the surrounding magnetic field [190]. If we assume that in neighboring rods, the cryptochromes are oriented at an angle of 0° and 90° relative to each other, than we obtain a “two-axis magnetometer” capable of perceiving changes in the induction of the surrounding field in space. Further processing of information received from cryptochromes occurs with the participation of the nervous system, according to the opponent process, as in the case of color vision of vertebrates and polarization vision of insects [192].

One of the most studied molecular mechanisms of magnetoreception is the cryptochrome signaling system. Cryptochromes are a group of dimeric flavoproteins of plants and animals that are sensitive to blue light (~430 nm), providing regulation of circadian rhythms and responses to changes in the magnetic field [193]. The magnetic sense of vertebrates, the fruit fly *Drosophila melanogaster* and the monarch butterfly *Danaus plexippus*, is light-dependent and mediated by the ultraviolet (UV)-A/blue light photoreceptor cytochromes 1 and 2 (cry 1, cry2). Cryptochromes are transcriptionally repressive signaling molecules and require UV-A/blue light (wavelength is below 420 nm) for magnetic field detection. It was previously assumed that the so-called tryptophan triad in the cryptochrome molecule is involved in light-sensitive magnetoreception and mediates the ability of cry to perceive the magnetic field. However, genetic engineering methods have shown that this is not the case and animal cry mediates light-dependent magnetoreception through an unconventional photochemical mechanism [194]. The expression of cryptochromes in the retina and brain of migratory birds, as well as their close connection with the processing of visual information and behavior, has been experimentally demonstrated [195]. There is evidence in the literature about the participation of cryptochromes in the magnetic-dependent regulation of plant growth and development [95]. Mutations in cry1 and cry2 abolished the effect of the magnetic field [94]. There are several types and classes of cryptochromes depending on the systematic affiliation of the organism and the function performed [196]. A cryptochrome, like chlorophyll, is capable of forming a photoinduced radical pair in vivo [150]. The idea that cryptochromes are the main target for magnetic fields was developed in numerous experiments with plants. The expression of gibberellin phytohormones during flowering of Arabidopsis was suppressed in HMC as compared to HMP [31]. However, no such suppression was observed in cryptochrome mutant plants (cry1/cry2). In another study by the same authors, the delay in flowering in Arabidopsis in HMC was explained by changes in auxin distribution and increased cryptochrome-dependent expression of transcription repressor genes [95]. Suppression of many genes during *Arabidopsis* growth in HMC was also observed [90,96]. A blue-light-dependent cry1 and cry2 phosphorylation rate increased in a magnetic field of 500 μTl and decreased in HMC [102]. Similar effects of magnetic fields on the phosphorylation of the C-terminal domain of a cryptochrome and the key role of flavin reoxidation in this process were found [197,198]. The data obtained in these studies confirm the participation of cryptochromes and the involvement of the quantum theory RPM in the development of magnetobiological effects [150]. Cryptochromes were first described in *Arabidopsis thaliana* in work [199]. Cryptochromes are flavoproteins that have involvement in circadian rhythms to hormonal DNA signaling [200]. A cryptochrome binds to a photolyase, and flavin adenine dinucleotide (FAD), which, in conjunction with a nearby tryptophan residue, plays a key role in the RPM mechanism of magnetoreception by forming a radical bundle (FAD-TrpH^+^) [150].

However, manifestations of magnetobiological effects in the dark have been described in the literature [90,101,102]. This suggests that the magnetosensitive stage of the reaction may be a reoxidation step with superoxide radicals (FADH• O_2_•^−^) [201,202]. Flavin semiquinone, superoxide, and radical scavenger are considered to be a single radical triad system that plays a crucial role in the magnetosensitivity of a cryptochrome [201].

The singlet–triplet conversion in radicals is sensitive to the direction of the magnetic field, so the radical pair may provide the functioning of animal cryprochrome-dependent magnetic compasses [150]. The RPM is known to have low sensitivity. In a single radical pair, the GMF produces a magnetic effect that is unlikely to exceed 0.1% of the baseline, and the expected chemical yield is negligible [156]. However, in living nature, there is a mechanism for increasing the sensitivity of radical pairs: numerous duplications and ordered arrangement. Thus, the responses of all radical pairs are summed up and reach a sufficient amplitude to trigger signaling cascades (in the central nervous system in animals or transcriptional regulation in plants). For the eyes of vertebrates and insects, the signal-to-noise ratio can reach ~1000 [203,204]. In plant cells, cryptochromes are localized in an orderly manner, which also suggests the role of ordering of cryptochromes in signal amplification [205].

It is noteworthy that similar opsin structures were found in mice under low-light conditions [191], and birds often migrate at night, which indirectly confirms that a similar structure may be involved in night orientation using magnetoreception. The estimated number of photons incident on one photoreceptor at night varies from 1 photon/s (cloudy moonless night), to 10^3^ photons/s (cloudy moonlit night), and 10^5^ photons/s (clear weather) [206,207]. According to calculations, a value of 1 photon/s is too low to implement the magnetoreception mechanism with the participation of radical pairs, but a value of the order of >10^3^ photons/s may be sufficient [150]. Interestingly, birds prefer to migrate in clear weather, above or below clouds [208], indicating a requirement of a luminous flux of at least 10^3^ photons/s per photoreceptor. Consequently, the mechanism of radical pairs can theoretically be implemented during magnetoreception. There are suggestions about additional localization of cryptochromes in the retinal ganglia, but no ordered structures of “candidates” for the role of magnetoreceptors have been found [209,210].

As mentioned earlier, in addition to cryptochromes, a radical pair was found in the chlorophyll molecule [181]. In the bacteria *Rhodopseudomonas sphaeroides*, a dependence of the rate of photosynthesis on the induction of a magnetic field was discovered, and the functioning of a radical pair was suggested [154]. The formation of a radical pair involves the amino acid residue of tryptophan (tryptophan triad) and NADH of the active center of the protein. A photon of UV blue light is absorbed by NAD, and NAD loses H^+^ and gains an unpaired electron. Thus, a pair of radicals with opposite charges (NAD^•−^, tryptophan^•+^) and antiparallel spins (singlet state) is formed. An external magnetic field can cause a radical pair to transition to the triplet state (parallel spins). In the state of parallel spins, NAD can attach the missing H^+^, which is accompanied by conformational rearrangements of cryptochromes, triggering signaling cascades with the further release of signaling molecules, in the case of birds, and neurotransmitters [150].

In addition to cryptochromes, photolyases 4–6, which have a longer lifetime of the photoinduced radical pair FAD-tryptophan, can participate in magnetoreception in animals [211]. The lifetime of the stable state of the FAD-tryptophan radical pair ranges from 1 ns to 1 μs, which significantly exceeds the time of light effects [212]. For chlorophyll, the lifetime of the radical pair is several picoseconds [213]. Therefore, magnetoreception through cryptochromes is not only a photoinduced process. In addition, cryptochrome-independent mechanisms of magnetosensitivity have been described in plants [101].

Potential magnetoreceptors can be magnetic nanoparticles distributed in the body [140,213]. As a rule, they are nanoparticles of magnetite (Fe_3_O_4_) and/or maghemite (γ-Fe_2_O_3_). Such nanoparticles have been found in many organisms from bacteria [214] to humans [215]. Different types of magnetotactic bacteria use nanoparticles to orientate themselves in space [216,217]. For this purpose, nanoparticles are assembled inside special organelles named magnetosomes [218]. In addition, magnetosomes are used to store excess metals in bacteria. Metals storage is considered a primary function of magnetosomes [219]. About several dozen special genes (*mam* and *mms*) participate in the formation of magnetosomes and about 300 more enhance transcription in the process of magnetosome formation in the cell [220].

The content of iron nanoparticles in eukaryotes depends on organs or tissue [221]. For example, nanoparticles have been found in human brain cells at concentrations ranging from 10 to 100 ng/g for different sites [222]. It is most likely that magnetosomes are of a biogenic origin; that is, they are formed over time as a result of crystallization directly in the cellular environment since crystals 50 to 200 nm in size will not be able to cross the blood–brain barrier [223].

A magnetic nanoparticle can change the rate of spin-dependent chemical reactions under the influence of external magnetic fields [224]. The limit of sensitivity to magnetic fields with induction of the order of GMP for magnetic nanoparticles is about 2 degrees [225], which can provide good accuracy for navigation of migrating birds and animals [225,226,227,228]. For birds, an area at the base of the beak has been found in which an increased content of HF has been found [229]. This “organ” may be involved in navigation. This assumption is strengthened because, in addition to the direction of HMP (an analog of a compass), birds sense the vertical declination of magnetic fields.

The complex of MagR protein and cry molecules is considered to be a new type of magnetic sensor [230]. The MagR protein may be associated with magnetoreception in non-bacterial organisms, including birds and other animals. The mechanism of action of the MagR/cry complex is based on its presumed ability to rotate when the direction of the surrounding magnetic field changes. The MagR gene (*CG8198*) was discovered through analytical means in *Drosophila* [231] and its protein has since been observed and isolated in other organisms such as butterflies, pigeons, and humans. The MagR protein is classified as an iron–sulfur protein. Proteins containing iron, particularly those that produce iron–sulfur compounds, play an indispensable role in electron transport. Together with a cryptochrome (cry), it is posited that the MagR protein may function as a light-dependent compass of animals [230]. While researchers have presented convincing evidence that large complexes (~20 nm) of MagR and cry molecule complexes can rotate when subjected to fields of geomagnetic order, there remains a physical criticism of their work [232]. It has been observed that MagR/cry complexes containing only 40 iron atoms cannot possess a constant dipole moment. In contrast, the minimum size of magnetite nanoparticles possessing a magnetic moment is approximately 30 nm [233], and such particles contain ~10^6^ iron atoms. If we assume that these 40 atoms possess a solitary magnetic moment, their energy in a magnetic field would be five orders of magnitude lower than the energy of *kT*.

## 6. Dependence of Biological Effect Magnitude on Quantitative Characteristics of HMC

**Figure 7:**
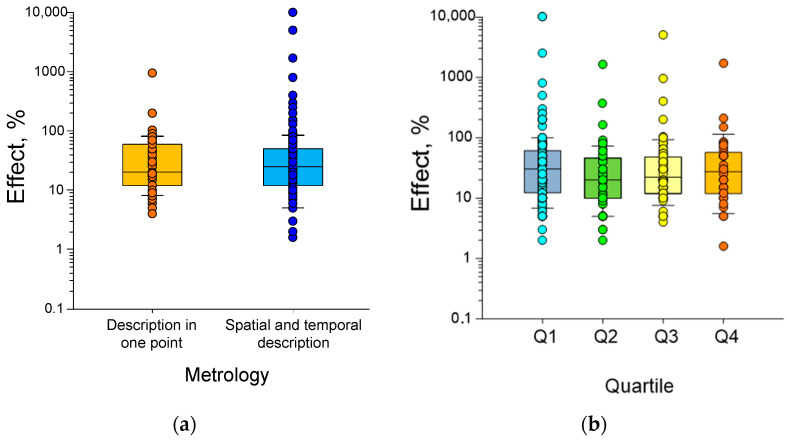
The distribution of biological effect values is dependent on methodology (**a**) and journal rating (**b**). Metrology in this case included HMC homogeneity analysis: measure of magnetic flux in one time and spatial point without variation description (**left**) or measure of magnetic flux in serial time and spatial points with variation description (**right**). Journal rating was based on SJR and actual quartiles (taken from https://www.scimagojr.com/journalrank.php, accessed on 25 October 2023). Each point is an experimental value from an analyzed article. The effects were calculated as the ratio of the difference between the values of the investigated parameter in HMC and Sham control and the value in Sham control. The result was expressed as a percentage. Percentage values were taken modulo. The total number of analyzed experimental points is 350. The results are shown as medians (box **centers**) with percentiles 25% and 50% (box **bottom** and **top**) and percentiles 10% and 90% (bars).

We attempted to estimate the dependence of the expression of biological effects of HMC on experimental conditions based on the data of the selected publications (Table 1). We analyzed quantitative data from 70 papers published during the last 30 years in journals of different ratings. Preliminarily, we evaluated the contribution of the features of the research methodology described in the papers, the applied statistical methods, and the rating of the journal in which the manuscript was published. As criteria for evaluating the methodology, we used the nature of the description of the magnetic field at HMC: evaluation of the temporal and spatial variation of the magnetic field induction at HMC and Sham conditions, and the availability of a 3D map of the magnetic field induction distribution inside the experimental setup (Figure 7a). The type of the applied criterion was used as an assessment of the statistical processing (Student’s *t*-test, Mann–Whitney U-test, etc.), analysis of variance (ANOVA), post hoc tests, and description of the tests of applicability of this or that statistical method. The criterion was applied to evaluate the Scientific Journal Rating (SJR) of the journal containing a publication (https://www.scimagojr.com/journalrank.php, accessed on 25 October 2023) and the actual quartile (Figure 7b).

We expected that in articles with a less detailed magnetic field homogeneity description and statistical methods, the severity of the effects shown would differ from works with more detailed descriptions. In addition, we expected that the magnitude of biological effects might depend in some way on the rating of the publication that published the study. A rating was considered an integral indicator of the quality of work. Of course, we realize that this division is conditional and the journal can change quartiles and move from Q1 to Q3, not due to a decrease in the quality of publications, but due to a decrease in funding in this area and as a consequence a decrease in the community. This does not make the articles published in this journal less qualitative [86,89].

However, we did not find any significant differences between the severity of biological effects in different groups of analyzed works, identified by the characteristics of the description of metrology, the rating of publications, and the details of the description of statistical methods. It is likely that the authors of a significant part of the works we analyzed fulfilled all the necessary metrological, methodological, and statistical conditions to obtain high-quality and reproducible results, but did not always describe them in detail.

**Figure 8:**
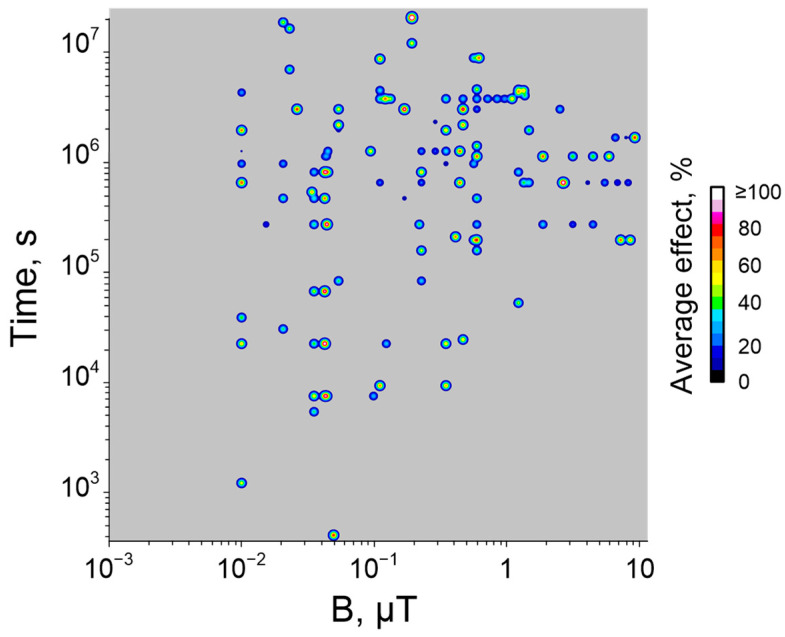
Distribution of mean values of biological effects by induction (B) and duration. The effect is calculated as the ratio of the difference between the values of the investigated parameter in GMU and Sham control and the value in Sham control. The result was expressed as a percentage. Percentage values were taken modulo.

The next step was to assess the distribution of the magnitude of the biological effect of HMC from magnetic field induction and exposure duration (Figure 8). The tendency was observed that the lower the magnetic field induction needed during HMC, the shorter the time to induce a biological effect. At the same time, we found that the magnitude of biological effects depended very little on time. High effects (>100%, in other words, a change of two or more times) were observed at times of the order of an hour (3.6 × 10^3^ s), a day (8.64 × 10^5^ s), and a month (~2.6 × 10^6^ s). Therefore, for a further analysis, we used only data on magnetic field induction during HMC.

**Figure 9:**
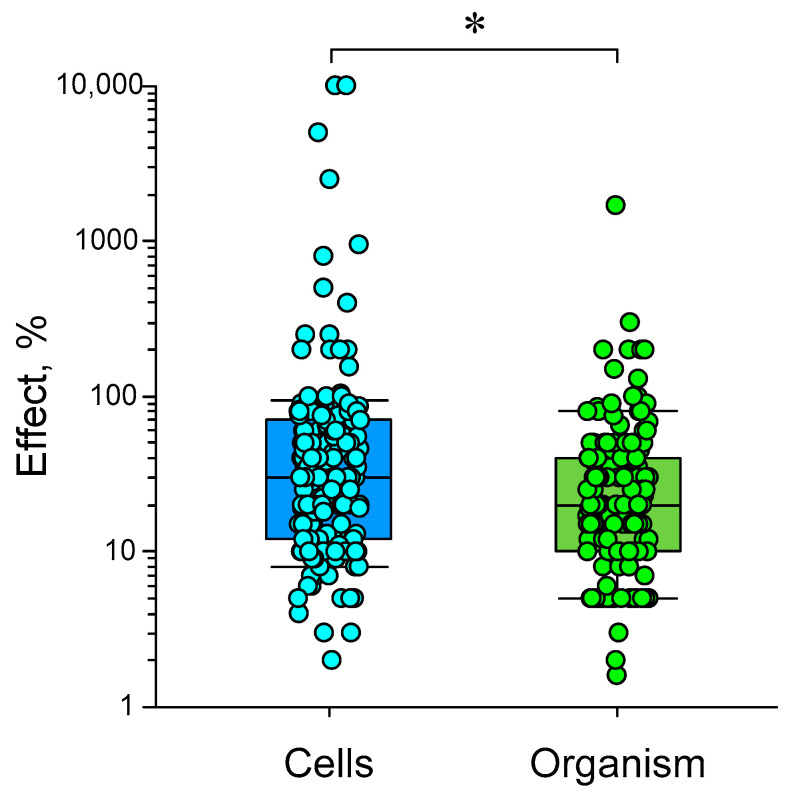
The distribution of HMC biological effects at the cell level depends on the object of study: cells (**left**) and organisms (organisms). The effects were calculated as the ratio of the difference between the values of the investigated parameter in HMC and Sham control and the value in Sham control. Absolute values of relative effects in papers are presented as the “Effect, %”. The total number of analyzed experimental points is 350. “*”—*p*-level < 0.05. Mann–Whitney rank sum test was used.

**Figure 10:**
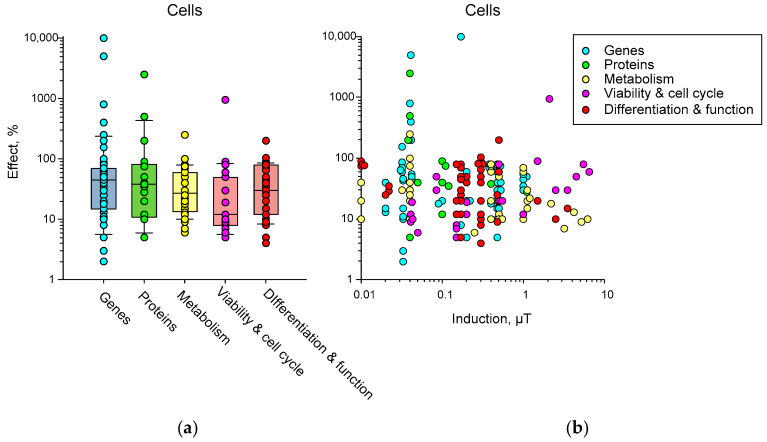
The distribution of HMC biological effects at the cell level depends on the object of study. (**a**) Box plots of the general distribution of the biological effect between different groups. The results are shown as medians (box **centers**) with percentiles 25% and 50% (box **bottom** and **top**) and percentiles 10% and 90% (bars), (**b**) dot plots of distribution of biological effect in different groups depending on magnetic field induction. Colors show different objects: cyan—gene expression change; green—protein concentration and enzyme activity; yellow—concentrations of metabolites and other biologically active compounds, and mitochondria functions; magenta—cell survival, proliferation rate, and distribution between cell circle phases; red—cell morphology, differentiation marker surface expression, migration, adhesion, specific electrical responses, etc. The effects were calculated as the ratio of the difference between the values of the investigated parameter in HMC and Sham control and the value in Sham control. Absolute values of relative effects in papers are presented as the “Effect, %”. Total number of analyzed experimental points is 350.

In the last stage, we divided the described-in-literature effects by the level of organization of life. We conditionally divided all effects into two groups: molecular–cellular and organ–organismal (Figure 9). We found that the effects of HMC were greater at the cellular level compared to the organismal level. The result obtained is an indirect confirmation of the fact that the contribution of non-specific mechanisms of biological effects of the magnetic field is higher in the case of cells compared to the whole organism. Within each level, we conditionally divided the results according to the types of methods used. Gene expression change, protein concentration, enzyme activity, concentrations of metabolites and mitochondria functions, cell survival and proliferation (rate, distribution between cell circle phases), and cell differentiation (marker surface expression, migration, adhesion, specific electrical responses, etc.) were chosen for the cell level. We found that in many cases, HMC has a greater effect on gene expression, protein concentration, and activity compared to other assessed parameters (Figure 10). Two explanations can be given for this fact. The first is that it is the “effect of low base”. That is, gene expression is too low in the control, and even a small absolute increase in expression leads to large relative effects. The second possible reason is that these methods capture the primary effects of MF receptions, which are “blurred” at the organism level.

**Figure 11:**
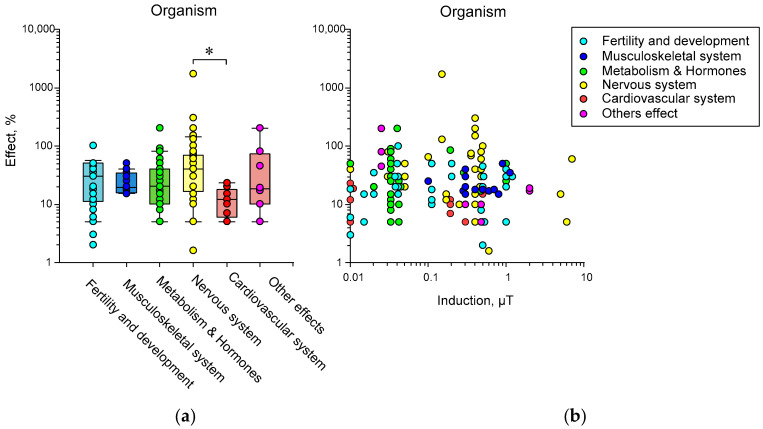
The distribution of HMC biological effects at the organism level depends on the object of study. (**a**) Box plots of the general distribution of the biological effect between different groups. The results are shown as medians (box **centers**) with percentiles 25% and 50% (box **bottom** and **top**) and percentiles 10% and 90% (bars), (**b**) dot plots of the distribution of biological effects in different groups depending on magnetic field induction. Colors show different objects: cyan—quantity and quality of offspring, speed of growth and development, size of adults; blue—structure and function of muscles and bones; green—concentrations of metabolites, microelements, hormones, and other biologically active compounds (measured in whole organ or organism); yellow—brain’s structure and behavior test results; red—heartbeat rate, microcirculation rate; magenta—survival, regeneration, etc. The effects were calculated as the ratio of the difference between the values of the investigated parameter in HMC and Sham control and the value in Sham control. Absolute values of relative effects in papers are presented as the “Effect, %”. The total number of analyzed experimental points is 350. “*”—*p*-level < 0.05. Kruskal–Wallis one-way ANOVA on ranks and Dunnett’s post hoc test were used.

The discovered tendency for the biological effects of HMC to increase upon transition to the molecular level is consistent with the ideas about the non-specificity of the action of magnetic fields and HMC [187]. As mentioned above, in some cases, changing magnetic conditions can affect individual molecular targets, but for the effect to manifest at a higher level of organization, an amplification cascade is required, as is the case with avian cryptochromes [192,194,204,234]. There appear to be no such amplification cascades for HMC, so at the more complex level of whole-cell responses, the biological effects of HMC are somewhat lower. It is noteworthy that this trend is observed only at induction values < 0.3 μT or less (Figure 11b).

At the organ–organism level, we found that HMCs have a greater effect on the functioning of the central nervous system, including behavioral responses and cognitive tests, than on the functioning of the cardiovascular system (Figure 11). This fact is interesting since the central nervous system is physiologically associated with the regulation of heart rate and immunity [235,236,237]. We assume that targets for the nonspecific action of the magnetic field may be present in the nervous system, for example, cryptochromes expressed in neurons or photolyases 4–6 [238,239]. The findings from our examination of increased sensitivity in the nervous system align with those of a recently published review [28]. On the other hand, the cardiovascular system is one of the most stable in the body. Large changes in the ECG, blood pressure, or heart rate indicate irreversible effects on the organism. Therefore, large relative effects of MF are unlikely there.

## 7. Conclusions

Hypomagnetic conditions (HMCs) have diverse and multidirectional effects on animals, plants, and bacteria. Parameters assessed include molecular processes (enzyme activity, translation regulation, mitochondrial function, protein self-assembly, etc.), changes in cells and tissues (morphology, proliferation, differentiation), as well as characteristics of the organism as a whole (behavior, survival, and fertility). Most effects are significant and, when quantified, amount to 10–30%. The vast majority of biological effects in HMC occur at inductions below 300 nT. Classical approaches for modeling HMC are shielding with soft magnetic materials and compensation using a system of Helmholtz coils. The latter are becoming more popular due to their accessibility and greater ability to control experimental conditions due to an active feedback system. Almost ten mechanisms of interaction of the magnetic field with living organisms have been theoretically described. Among them, the following mechanisms are the most applicable to HMCs: the influence of MFs on radical pairs, interaction with rotating molecules possessing magnetic moments, and interference quantum mechanisms. The RPM seems to be used in navigation by some animals and birds. This mechanism is currently the most confirmed. However, some physical assumptions in the RPM mechanism make it necessary to look for new mechanisms, such as LMMs. The latter is a specific mechanism since a single act of magnetoreception is enhanced by several orders of magnitude due to the number and uniform arrangement of cryptochromes. The effects of HMC at the cellular level are more pronounced than at the organismal level. This phenomenon indirectly confirms that the primary mechanisms of induction of magneto-biological effects are nonspecific. At the organismal level, the nervous system has the greatest sensitivity to HMC. Clarification of the molecular targets of nonspecific mechanisms and the search for ways of protection, a detailed assessment of the positive and negative effects of HMC, as well as the search for ways to minimize the adverse consequences of staying in HMC are the future tasks of magnetobiology and other related sciences.

## Data Availability

The raw data supporting the conclusions of this article will be made available by the authors, without undue reservation.

## References

[1] Panovska S., Korte M., Constable C.G. (2019). One Hundred Thousand Years of Geomagnetic Field Evolution. Rev. Geophys..

[2] Buddhi D., Singh R., Gehlot A. (2021). Magnetic Field in the Solar System—A Brief Review. Global Emerging Innovation Summit (GEIS-2021).

[3] Erdmann W., Kmita H., Kosicki J.Z., Kaczmarek Ł. (2021). How the Geomagnetic Field Influences Life on Earth—An Integrated Approach to Geomagnetobiology. Orig. Life Evol. Biosph..

[4] Finlay C.C., Maus S., Beggan C.D., Bondar T.N., Chambodut A., Chernova T.A., Chulliat A., Golovkov V.P., Hamilton B., Hamoudi M. (2010). International Geomagnetic Reference Field: The eleventh generation. Geophys. J. Int..

[5] Curto J.J. (2020). Geomagnetic solar flare effects: A review. J. Space Weather Space Clim..

[6] Herrmann F., Vorbach T. (2020). The geodynamo for non-geophysicists. Eur. J. Phys..

[7] Yamazaki Y. (2022). Solar and lunar daily geomagnetic variations and their equivalent current systems observed by Swarm. Earth Planets Space.

[8] Morozova A., Rebbah R. (2021). Comparison of the solar variations of the geomagnetic field at the Coimbra Magnetic Observatory (COI) obtained by different methods: Effect of the solar and geomagnetic activity. arXiv.

[9] Steinhilber F., Abreu J.A., Beer J., McCracken K.G. (2010). Interplanetary magnetic field during the past 9300 years inferred from cosmogenic radionuclides. J. Geophys. Res. Space Phys..

[10] Mo W.-c., Liu Y., He R. (2012). A Biological Perspective of The Hypomagnetic Field: From Definition Towards Mechanism. Prog. Biochem. Biophys..

[11] Berguig M.S., Hamoudi M., Lemouël J.L., Cohen Y. (2011). Validate global mapping of internal lunar magnetic field. Arab. J. Geosci..

[12] Cain J.C., Ferguson B.B., Mozzoni D. (2003). An n = 90 internal potential function of the Martian crustal magnetic field. J. Geophys. Res. Planets.

[13] Sinčák M., Sedlakova-Kadukova J. (2023). Hypomagnetic Fields and Their Multilevel Effects on Living Organisms. Processes.

[14] Vidotto A.A. (2021). The evolution of the solar wind. Living Rev. Sol. Phys..

[15] Lammer H., Bredehöft J.H., Coustenis A., Khodachenko M.L., Kaltenegger L., Grasset O., Prieur D., Raulin F., Ehrenfreund P., Yamauchi M. (2009). What makes a planet habitable?. Astron. Astrophys. Rev..

[16] Tarduno J.A., Cottrell R.D., Watkeys M.K., Hofmann A., Doubrovine P.V., Mamajek E.E., Liu D., Sibeck D.G., Neukirch L.P., Usui Y. (2010). Geodynamo, Solar Wind, and Magnetopause 3.4 to 3.45 Billion Years Ago. Science.

[17] Michalski G., Bhattacharya S.K., Girsch G. (2014). NOx cycle and the tropospheric ozone isotope anomaly: An experimental investigation. Atmos. Chem. Phys..

[18] Tarduno J.A., Blackman E.G., Mamajek E.E. (2014). Detecting the oldest geodynamo and attendant shielding from the solar wind: Implications for habitability. Phys. Earth Planet. Inter..

[19] Wiltschko R., Nießner C., Wiltschko W. (2021). The Magnetic Compass of Birds: The Role of Cryptochrome. Front. Physiol..

[20] Kirschvink J.L., Jones D.S., MacFadden B.J. (2013). Magnetite Biomineralization and Magnetoreception in Organisms: A New Biomagnetism.

[21] Obleser P., Hart V., Malkemper E.P., Begall S., Holá M., Painter M.S., Červený J., Burda H. (2016). Compass-controlled escape behavior in roe deer. Behav. Ecol. Sociobiol..

[22] Hart V., Malkemper E.P., Kušta T., Begall S., Nováková P., Hanzal V., Pleskač L., Ježek M., Policht R., Husinec V. (2013). Directional compass preference for landing in water birds. Front. Zool..

[23] Bianco G., Köhler R.C., Ilieva M., Åkesson S. (2019). Magnetic body alignment in migratory songbirds: A computer vision approach. J. Exp. Biol..

[24] Smith M.J., Sheehan P.E., Perry L.L., O’Connor K., Csonka L.N., Applegate B.M., Whitman L.J. (2006). Quantifying the Magnetic Advantage in Magnetotaxis. Biophys. J..

[25] Uebe R., Schüler D. (2016). Magnetosome biogenesis in magnetotactic bacteria. Nat. Rev. Microbiol..

[26] Begall S., Červený J., Neef J., Vojtěch O., Burda H. (2008). Magnetic alignment in grazing and resting cattle and deer. Proc. Natl. Acad. Sci. USA.

[27] Davies E. (2023). The decrease in diurnal oxygen production in Elodea under the influence of high geomagnetic variability: The role of light, temperature and atmospheric pressure. Int. J. Biometeorol..

[28] Zhang Z., Xue Y., Yang J., Shang P., Yuan X. (2021). Biological Effects of Hypomagnetic Field: Ground-Based Data for Space Exploration. Bioelectromagnetics.

[29] Tombarkiewicz B. (2008). Effect of long-term geomagnetic field deprivation on the concentration of some elements in the hair of laboratory rats. Environ. Toxicol. Pharmacol..

[30] Fu J.-P., Mo W.-C., Liu Y., Bartlett P.F., He R.-Q. (2016). Elimination of the geomagnetic field stimulates the proliferation of mouse neural progenitor and stem cells. Protein Cell.

[31] Xu C., Yu Y., Zhang Y., Li Y., Wei S. (2017). Gibberellins are involved in effect of near-null magnetic field on Arabidopsis flowering. Bioelectromagnetics.

[32] Wang X.K., Ma Q.F., Jiang W., Lv J., Pan W.D., Song T., Wu L.-F. (2008). Effects of Hypomagnetic Field on Magnetosome Formation ofMagnetospirillum MagneticumAMB-1. Geomicrobiol. J..

[33] Grinberg M.A., Vodeneev V.A., Il’in N.V., Mareev E.A. (2023). Laboratory Simulation of Photosynthesis in a Wide Range of Electromagnetic and Radiation Environment Parameters. Astron. Rep..

[34] Martino C.F., Perea H., Hopfner U., Ferguson V.L., Wintermantel E. (2010). Effects of weak static magnetic fields on endothelial cells. Bioelectromagnetics.

[35] Wang D.L., Wang X.S., Xiao R., Liu Y., He R.Q. (2008). Tubulin assembly is disordered in a hypogeomagnetic field. Biochem. Biophys. Res. Commun..

[36] Beischer D.E. (1971). The null magnetic field as reference for the study of geomagnetic directional effects in animals and man. Ann. N. Y. Acad. Sci..

[37] Katiukhin L.N. (2019). Rheological properties of the erythrocytes in weakened static magnetic field of the earth in vitro study. J. Sci. Res. Rep..

[38] Erdmann W., Idzikowski B., Kowalski W., Kosicki J.Z., Kaczmarek Ł. (2021). Tolerance of two anhydrobiotic tardigrades Echiniscus testudo and Milnesium inceptum to hypomagnetic conditions. PeerJ.

[39] Zhang H.-T., Zhang Z.-J., Mo W.-C., Hu P.-D., Ding H.-M., Liu Y., Hua Q., He R.-Q. (2017). Shielding of the geomagnetic field reduces hydrogen peroxide production in human neuroblastoma cell and inhibits the activity of CuZn superoxide dismutase. Protein Cell.

[40] Mo W.-C., Liu Y., Cooper H.M., He R.-Q. (2012). Altered development of Xenopus embryos in a hypogeomagnetic field. Bioelectromagnetics.

[41] Beischer D.E., Miller II E.F., Knepton J.C. (1967). Exposure of Man to Low Intensity Magnetic Fields in a Coil System.

[42] Binhi V.N., Sarimov R.M. (2009). Zero magnetic field effect observed in human cognitive processes. Electromagn. Biol. Med..

[43] Sarimov R.M., Binhi V.N., Milyaev V.A. (2008). The influence of geomagnetic field compensation on human cognitive processes. Biophysics.

[44] Gurfinkel Y.I., At’kov O.Y., Vasin A.L., Breus T.K., Sasonko M.L., Pishchalnikov R.Y. (2016). Effect of zero magnetic field on cardiovascular system and microcirculation. Life Sci. Space Res..

[45] Wan G.-J., Jiang S.-L., Zhao Z.-C., Xu J.-J., Tao X.-R., Sword G.A., Gao Y.-B., Pan W.-D., Chen F.-J. (2014). Bio-effects of near-zero magnetic fields on the growth, development and reproduction of small brown planthopper, Laodelphax striatellus and brown planthopper, Nilaparvata lugens. J. Insect Physiol..

[46] Sarimov R., Binhi V. (2020). Low-Frequency Magnetic Fields in Cars and Office Premises and the Geomagnetic Field Variations. Bioelectromagnetics.

[47] Mo W.-C., Zhang Z.-J., Wang D.-L., Liu Y., Bartlett P.F., He R.-Q. (2016). Shielding of the Geomagnetic Field Alters Actin Assembly and Inhibits Cell Motility in Human Neuroblastoma Cells. Sci. Rep..

[48] Krylov V., Machikhin A., Sizov D., Guryleva A., Sizova A., Zhdanova S., Tchougounov V., Burlakov A. (2022). Influence of hypomagnetic field on the heartbeat in zebrafish embryos. Front. Physiol..

[49] Mo W., Liu Y., Bartlett P.F., He R. (2014). Transcriptome profile of human neuroblastoma cells in the hypomagnetic field. Sci. China Life Sci..

[50] Wang G.-M., Fu J.-P., Mo W.-C., Zhang H.-T., Liu Y., He R.-Q. (2022). Shielded geomagnetic field accelerates glucose consumption in human neuroblastoma cells by promoting anaerobic glycolysis. Biochem. Biophys. Res. Commun..

[51] Ogneva I.V., Usik M.A., Burtseva M.V., Biryukov N.S., Zhdankina Y.S., Sychev V.N., Orlov O.I. (2020). Drosophila melanogaster Sperm under Simulated Microgravity and a Hypomagnetic Field: Motility and Cell Respiration. Int. J. Mol. Sci..

[52] Gurfinkel Y.I., Vasin A.L., Pishchalnikov R.Y., Sarimov R.M., Sasonko M.L., Matveeva T.A. (2018). Geomagnetic storm under laboratory conditions: Randomized experiment. Int. J. Biometeorol..

[53] Pishchalnikov R.Y., Gurfinkel Y.I., Sarimov R.M., Vasin A.L., Sasonko M.L., Matveeva T.A., Binhi V.N., Baranov M.V. (2019). Cardiovascular response as a marker of environmental stress caused by variations in geomagnetic field and local weather. Biomed. Signal Process. Control.

[54] Belova N.A., Lednev V.V. (2000). Dependence of gravitotropic reaction in segments of flax stems on frequency and amplitude of variable components of a weak combined magnetic field. Biophysics.

[55] Belova N.A., Ermakov A.M., Znobishcheva A.V., Srebnitskaya L.K., Lednev V.V. (2010). The influence of extremely weak alternating magnetic fields on the regeneration of planarians and the gravitropic response of plants. Biophysics.

[56] Canova A., del Pino López J., Giaccone L., Manca M. (2015). Active Shielding System for ELF Magnetic Fields. IEEE Trans. Magn..

[57] Khodanovich M.Y., Krivova N.A., Gull Y.V., Zelenskaya A.Y., Bondartseva N.V. (2011). Effects of long-term geomagnetic field deprivation on bioelectrical activity of brain of laboratory rats. Tomsk. State Univ. J..

[58] Khodanovich M.Y., Gul E.V., Zelenskaja A.E., Pan E.S., Krivova N.A. (2013). Effect of long-term geomagnetic field weakening on aggressiveness of rats and opioidergic neurons activation. Tomsk. State Univ. J. Biol..

[59] Zhang X., Li J.-F., Wu Q.-J., Li B., Jiang J.-C. (2007). Effects of hypomagnetic field on noradrenergic activities in the brainstem of golden hamster. Bioelectromagnetics.

[60] Zhang B., Wang L., Zhan A., Wang M., Tian L., Guo W., Pan Y. (2021). Long-term exposure to a hypomagnetic field attenuates adult hippocampal neurogenesis and cognition. Nat. Commun..

[61] Tian L., Luo Y., Zhan A., Ren J., Qin H., Pan Y. (2022). Hypomagnetic Field Induces the Production of Reactive Oxygen Species and Cognitive Deficits in Mice Hippocampus. Int. J. Mol. Sci..

[62] Wang X., Xu M., Li B., Li D., Jiang J. (2003). Long-term memory was impaired in one-trial passive avoidance task of day-old chicks hatching from hypomagnetic field space. Chin. Sci. Bull..

[63] Zhang B., Lu H., Xi W., Zhou X., Xu S., Zhang K., Jiang J., Li Y., Guo A. (2004). Exposure to hypomagnetic field space for multiple generations causes amnesia in Drosophila melanogaster. Neurosci. Lett..

[64] Zhang Y., Pan W. (2021). Removal or component reversal of local geomagnetic field affects foraging orientation preference in migratory insect brown planthopper Nilaparvata lugens. PeerJ.

[65] Xu J., Pan W., Zhang Y., Li Y., Wan G., Chen F., Sword G.A., Pan W. (2017). Behavioral evidence for a magnetic sense in the oriental armyworm, Mythimna separata. Biol. Open.

[66] Mannino G., Casacci L.P., Bianco Dolino G., Badolato G., Maffei M.E., Barbero F. (2023). The Geomagnetic Field (GMF) Is Necessary for Black Garden Ant (Lasius niger L.) Foraging and Modulates Orientation Potentially through Aminergic Regulation and MagR Expression. Int. J. Mol. Sci..

[67] Kantserova N.P., Krylov V.V., Lysenko L.A., Ushakova N.V., Nemova N.N. (2018). Effects of Hypomagnetic Conditions and Reversed Geomagnetic Field on Calcium-Dependent Proteases of Invertebrates and Fish. Izv. Atmos. Ocean. Phys..

[68] Wan G.J., Jiang S.L., Zhang M., Zhao J.Y., Zhang Y.C., Pan W.D., Sword G.A., Chen F.J. (2020). Geomagnetic field absence reduces adult body weight of a migratory insect by disrupting feeding behavior and appetite regulation. Insect Sci..

[69] Jia B., Xie L., Zheng Q., Yang P.F., Zhang W.J., Ding C., Qian A.R., Shang P.A. (2014). A Hypomagnetic Field Aggravates Bone Loss Induced by Hindlimb Unloading in Rat Femurs. PLoS ONE.

[70] Yang J., Meng X., Dong D., Xue Y., Chen X., Wang S., Shen Y., Zhang G., Shang P. (2018). Iron overload involved in the enhancement of unloading-induced bone loss by hypomagnetic field. Bone.

[71] Fesenko E.E., Mezhevikina L.M., Osipenko M.A., Gordon R.Y., Khutzian S.S. (2010). Effect of the “zero” Magnetic Field on Early Embryogenesis in Mice. Electromagn. Biol. Med..

[72] Wan G.-J., Yuan R., Wang W.-J., Fu K.-Y., Zhao J.-Y., Jiang S.-L., Pan W.-D., Sword G.A., Chen F.-J. (2016). Reduced geomagnetic field may affect positive phototaxis and flight capacity of a migratory rice planthopper. Anim. Behav..

[73] Yan M.-m., Zhang L., Cheng Y.-x., Sappington T.W., Pan W.-d., Jiang X.-f. (2021). Effect of a near-zero magnetic field on development and flight of oriental armyworm (Mythimna separata). J. Integr. Agric..

[74] Krylov V.V., Bolotovskaya I.V., Osipova E.A. (2013). The response of european *Daphnia magna* straus and australian *Daphnia carinata* king to changes in geomagnetic field. Electromagn. Biol. Med..

[75] Binhi V.N., Sarimov R.M. (2013). Effect of the hypomagnetic field on the size of the eye pupil. arXiv.

[76] Moissl-Eichinger C., Erdmann W., Idzikowski B., Kowalski W., Szymański B., Kosicki J.Z., Kaczmarek Ł. (2017). Can the tardigrade Hypsibius dujardini survive in the absence of the geomagnetic field?. PLoS ONE.

[77] Temour’yants N.A., Kostyuk A.S., Tumanyants K.N. (2011). Dynamics and Infradian Rhythmics of Thermal/Pain Sensitivity of the Helix Mollusc under the Action of Electromagnetic Fields. Neurophysiology.

[78] Burger T., Lucová M., Moritz R.E., Oelschläger H.H.A., Druga R., Burda H., Wiltschko W., Wiltschko R., Němec P. (2010). Changing and shielded magnetic fields suppress c-Fos expression in the navigation circuit: Input from the magnetosensory system contributes to the internal representation of space in a subterranean rodent. J. R. Soc. Interface.

[79] Gurfinkel Y.I., Vasin A.L., Matveeva T.A., Sasonko M.L. (2014). Evaluation of the hypomagnetic environment effects on capillary blood circulation, blood pressure and heart rate. Aviakosmicheskaia I Ekol. Meditsina Aerosp. Environ..

[80] Demin A.V., Suvorov A.V., Orlov O.I. (2021). Features of hemodynamics in healthy men under hypomagnetic conditions. Aviakosm. Ekolog. Med..

[81] Ciorba D., Morariu V.V. (2009). Life in zero magnetic field. Iii. Activity of aspartate aminotransferase and alanine aminotransferase during in vitro aging of human blood. Electro-Magnetobiology.

[82] Novikov V.V., Yablokova E.V., Fesenko E.E. (2018). The Effect of a “Zero” Magnetic Field on the Production of Reactive Oxygen Species in Neutrophils. Biophysics.

[83] Mo W.-C., Fu J.-P., Ding H.-M., Liu Y., Hua Q., He R.-Q. (2015). Hypomagnetic Field Alters Circadian Rhythm and Increases Algesia in Adult Male Mice. Prog. Biochem. Biophys..

[84] Nepomnyashchikh L.M., Lushnikova E.L., Klinnikova M.G., Molodykh O.P., Ashcheulova N.V. (1997). Effect of hypogeomagnetic field on tissue and intracellular reorganization of mouse myocardium. Bull. Exp. Biol. Med..

[85] Hu P., Mo W.-C., Fu J.-P., Liu Y., He R.Q. (2020). Long-term hypogeomagnetic field exposure reduces muscular mitochondrial function and exercise capacity in adult male mice. Prog. Biochem. Biophys..

[86] Fu J.-P., Mo W.-C., Liu Y., He R.-Q. (2016). Decline of cell viability and mitochondrial activity in mouse skeletal muscle cell in a hypomagnetic field. Bioelectromagnetics.

[87] Tsetlin V.V., Zotin A.A., Moisa S.S. (2014). Effect of altered magnetic field on the development of great ramshorn Planorbarius corneus (gastropoda, planorbidae). Aviakosm. Ekolog. Med..

[88] Mo W.-C., Zhang Z.-J., Liu Y., Zhai G.-J., Jiang Y.-D., He R.-Q. (2011). Effects of a hypogeomagnetic field on gravitropism and germination in soybean. Adv. Space Res..

[89] Xu C., Wei S., Lu Y., Zhang Y., Chen C., Song T. (2013). Removal of the local geomagnetic field affects reproductive growth inArabidopsis. Bioelectromagnetics.

[90] Agliassa C., Narayana R., Bertea C.M., Rodgers C.T., Maffei M.E. (2018). Reduction of the geomagnetic field delays *Arabidopsis thaliana* flowering time through downregulation of flowering-related genes. Bioelectromagnetics.

[91] Islam M., Maffei M.E., Vigani G. (2020). The Geomagnetic Field Is a Contributing Factor for an Efficient Iron Uptake in Arabidopsis thaliana. Front. Plant Sci..

[92] Narayana R., Fliegmann J., Paponov I., Maffei M.E. (2018). Reduction of geomagnetic field (GMF) to near null magnetic field (NNMF) affects Arabidopsis thaliana root mineral nutrition. Life Sci. Space Res..

[93] Negishi Y., Hashimoto A., Tsushima M., Dobrota C., Yamashita M., Nakamura T. (1999). Growth of pea epicotyl in low magnetic field implication for space research. Adv. Space Res..

[94] Agliassa C., Narayana R., Christie J.M., Maffei M.E. (2018). Geomagnetic field impacts on cryptochrome and phytochrome signaling. J. Photochem. Photobiol. B Biol..

[95] Xu C., Zhang Y., Yu Y., Li Y., Wei S. (2018). Suppression of Arabidopsis flowering by near-null magnetic field is mediated by auxin. Bioelectromagnetics.

[96] Xu C., Yin X., Lv Y., Wu C., Zhang Y., Song T. (2012). A near-null magnetic field affects cryptochrome-related hypocotyl growth and flowering in Arabidopsis. Adv. Space Res..

[97] Parmagnani A.S., Betterle N., Mannino G., D’Alessandro S., Nocito F.F., Ljumovic K., Vigani G., Ballottari M., Maffei M.E. (2023). The Geomagnetic Field (GMF) Is Required for Lima Bean Photosynthesis and Reactive Oxygen Species Production. Int. J. Mol. Sci..

[98] Harris S.-R., Henbest K.B., Maeda K., Pannell J.R., Timmel C.R., Hore P.J., Okamoto H. (2009). Effect of magnetic fields on cryptochrome-dependent responses in *Arabidopsis thaliana*. J. R. Soc. Interface.

[99] Vigani G., Islam M., Cavallaro V., Nocito F.F., Maffei M.E. (2021). Geomagnetic Field (GMF)-Dependent Modulation of Iron-Sulfur Interplay in Arabidopsis thaliana. Int. J. Mol. Sci..

[100] Dhiman S.K., Galland P. (2018). Effects of weak static magnetic fields on the gene expression of seedlings of Arabidopsis thaliana. J. Plant Physiol..

[101] Agliassa C., Maffei M.E. (2019). Reduction of geomagnetic field (GMF) to near null magnetic field (NNMF) affects some Arabidopsis thaliana clock genes amplitude in a light independent manner. J. Plant Physiol..

[102] Xu C., Lv Y., Chen C., Zhang Y., Wei S. (2014). Blue light-dependent phosphorylations of cryptochromes are affected by magnetic fields in Arabidopsis. Adv. Space Res..

[103] Dhiman S.K., Wu F., Galland P. (2023). Effects of weak static magnetic fields on the development of seedlings of Arabidopsis thaliana. Protoplasma.

[104] Martino C.F., Portelli L., McCabe K., Hernandez M., Barnes F. (2010). Reduction of the Earth’s magnetic field inhibits growth rates of model cancer cell lines. Bioelectromagnetics.

[105] Binhi V.N. (2002). Magnetobiology: Experiments and Models.

[106] Li J.F., Wu Q.J., Wang Q., Jiang J.C., Jin H.Q., Lin Y.F. (2001). Effect of magnetic free field space (MFFS) on GABA, glycine andtaurine of cortex, cerebellum and basilar nucleus in hamster. Prog. Biochem. Biophys..

[107] Baek S., Choi H., Park H., Cho B., Kim S., Kim J. (2019). Effects of a hypomagnetic field on DNA methylation during the differentiation of embryonic stem cells. Sci. Rep..

[108] Roemer K., Mo W.-c., Zhang Z.-j., Liu Y., Bartlett P.F., He R.-Q. (2013). Magnetic Shielding Accelerates the Proliferation of Human Neuroblastoma Cell by Promoting G1-Phase Progression. PLoS ONE.

[109] Eldashev I.S., Shchegolev B.F., Surma S.V., Belostotskaya G.B. (2011). Influence of low-intensity magnetic fields on the development of satellite muscle cells of a newborn rat in primary culture. Biophysics.

[110] Yang J., Zhang J., Ding C., Dong D., Shang P. (2018). Regulation of Osteoblast Differentiation and Iron Content in MC3T3-E1 Cells by Static Magnetic Field with Different Intensities. Biol. Trace Elem. Res..

[111] Juutilainen J., Herrala M., Luukkonen J., Naarala J., Hore P.J. (2018). Magnetocarcinogenesis: Is there a mechanism for carcinogenic effects of weak magnetic fields?. Proc. R. Soc. B Biol. Sci..

[112] Jagetia G.C., Martino C.F., Castello P.R. (2011). Modulation of Hydrogen Peroxide Production in Cellular Systems by Low Level Magnetic Fields. PLoS ONE.

[113] Robison J.G., Pendleton A.R., Monson K.O., Murray B.K., O’Neill K.L. (2002). Decreased DNA repair rates and protection from heat induced apoptosis mediated by electromagnetic field exposure. Bioelectromagnetics.

[114] Babych V.I. (1995). The characteristics of tissue lipid peroxidation in the internal organs and the lipid metabolic indices of the blood plasma in a low geomagnetic field. Fiziolohichnyi Zhurnal.

[115] Babych V.I. (1996). The characteristics of tissue lipid peroxidation of the internal organs in anaphylaxis under the action of a hypo- or hypermagnetic field. Fiziolohichnyi Zhurnal.

[116] Zhang Y., Zeng L., Wei Y., Zhang M., Pan W., Sword G.A., Yang F., Chen F., Wan G. (2022). Reliable reference genes for gene expression analyses under the hypomagnetic field in a migratory insect. Front. Physiol..

[117] Belyaev I.Y., Alipov Y.D., Harms-Ringdahl M. (1997). Effects of zero magnetic field on the conformation of chromatin in human cells. Biochim. Biophys. Acta (BBA)-Gen. Subj..

[118] Xue X., Ali Y.F., Liu C., Hong Z., Luo W., Nie J., Li B., Jiao Y., Liu N.-A. (2020). Geomagnetic Shielding Enhances Radiation Resistance by Promoting DNA Repair Process in Human Bronchial Epithelial Cells. Int. J. Mol. Sci..

[119] McCreary C.R., Dixon S.J., Fraher L.J., Carson J.J.L., Prato F.S. (2006). Real-time measurement of cytosolic free calcium concentration in Jurkat cells during ELF magnetic field exposure and evaluation of the role of cell cycle. Bioelectromagnetics.

[120] Zhang S.-D., Petersen N., Zhang W.-J., Cargou S., Ruan J., Murat D., Santini C.-L., Song T., Kato T., Notareschi P. (2014). Swimming behaviour and magnetotaxis function of the marine bacterium strain MO-1. Environ. Microbiol. Rep..

[121] Poiata A., Creanga D.E., Morariu V.V. (2009). Life in zero magnetic field. V. *E. coli* resistance to antibiotics. Electromagn. Biol. Med..

[122] Creanga D.E., Poiata A., Morariu V.V., Tupu P. (2004). Zero-magnetic field effect in pathogen bacteria. J. Magn. Magn. Mater..

[123] Ilyin V.K., Orlov O.I., Morozova Y.A., Skedina M.A., Vladimirov S.K., Plotnikov E.V., Artamonov A.A. (2022). Prognostic model for bacterial drug resistance genes horizontal spread in space-crews. Acta Astronaut..

[124] Lutz K., Cadiou H., Trevino T., Cinelli I. Electromagnetic fields to sustain life on earth, in apace, and planets. Proceedings of the 72nd International Astronautical Congress (IAC).

[125] Blagau A., Ersen A., Dobrescu C., Marghitu O. (2022). Astro Pi sensor onboard the International Space Station as magnetic field surveyor. Acta Astronaut..

[126] Pandey S., Garg T., Singh K., Rai S. (1996). Effect of magnetically induced water structure on the oestrous cycles of albino female mice Musmusculus. Electro-Magnetobiology.

[127] Rai S., Garg T., Vashistha H. (1996). Possible Effect of Magnetically Induced Water Structures on Photosynthetic Electron Transport Chains of a Green Alga Chlorella Vulgarts. Electro-Magnetobiology.

[128] Devyatkov N.D., Kislov V.Y., Kislov V.V., Kolesov V.V., Smirnov V.F., Chigin E.P. (1996). Detection of the effect of normalisation of the functional state of human internal organs under the influence of water activated by millimetre radiation. Millimetre Waves Biol. Med..

[129] Ružič R., Jerman I. (1998). Influence of Ca^2+^ in biological effects of direct and indirect ELF magnetic field stimulation. Electro-Magnetobiology.

[130] Novikov V.V., Yablokova E.V., Fesenko E.E. (2020). The Role of Water in the Effect of Weak Combined Magnetic Fields on Production of Reactive Oxygen Species (ROS) by Neutrophils. Appl. Sci..

[131] Colic M., Morse D. (1998). Mechanism of the long-term effects of electromagnetic radiation on solutions and suspended colloids. Langmuir.

[132] Lobyshev V.I. (2021). Evolution of High-Frequency Conductivity of Pure Water Samples Subjected to Mechanical Action: Effect of a Hypomagnetic Filed. Phys. Wave Phenom..

[133] Penkov N. (2020). Temporal dynamics of the scattering properties of deionized water. Phys. Wave Phenom..

[134] Konovalov A., Ryzhkina I., Maltzeva E., Murtazina L., Kiseleva Y., Kasparov V., Palmina N. (2015). Nanoassociate formation in highly diluted water solutions of potassium phenosan with and without permalloy shielding. Electromagn. Biol. Med..

[135] Xiao-Feng P., Gui-Fa S. (2013). The Changes of Physical Properties of Water Arising from the Magnetic Field and its Mechanism. Mod. Phys. Lett. B.

[136] Shen X. (2011). Increased dielectric constant in the water treated by extremely low frequency electromagnetic field and its possible biological implication. J. Phys. Conf. Ser..

[137] Belov A.A., Konyukhov V.K., Stepanov A.V. (1997). Fluctuations of water dielectric permittivity under thermal and mechanical effects on water. Short Commun. Phys. FIAN.

[138] Astashev M., Serov D., Sarimov R., Gudkov S. (2023). Influence of the Vibration Impact Mode on the Spontaneous Chemiluminescence of Aqueous Protein Solutions. Phys. Wave Phenom..

[139] Novikov V.V., Kuvichkin V.V., Fesenko E.E. (1999). Effect of weak combined low frequency constant and alternative magnetic fields on intrinsic fluorescence of proteins in aqueous solutions. Biophysics.

[140] Binhi V.N., Alipov Y.D., Belyaev I.Y. (2001). Effect of static magnetic field on E. coli cells and individual rotations of ion-protein complexes. Bioelectromagnetics.

[141] Demin A.V. (2022). Characteristics of healthy men hemodynamics in model martian hypomagnetic environment. Biomed. Radioelectron..

[142] Ferrone K., Willis C., Guan F., Ma J., Peterson L., Kry S. (2023). A Review of Magnetic Shielding Technology for Space Radiation. Radiation.

[143] Bhattacharjie A., Michael I. (1964). Mass and magnetic dipole shielding against electrons of the artificial radiation belt. AIAA J..

[144] Trukhanov K., Morozov D. (1970). Optimization of a Magnetic Radiation Shield. Sov. Phys. Tech. Phys..

[145] Barthel J., Sarigul-Klijn N. (2019). A review of radiation shielding needs and concepts for space voyages beyond Earth’s magnetic influence. Prog. Aerosp. Sci..

[146] Musenich R., Calvelli V., Giraudo M., Vuolo M., Ambroglini F., Battiston R. (2018). The Limits of Space Radiation Magnetic Shielding: An Updated Analysis. IEEE Trans. Appl. Supercond..

[147] Spillantini P., Casolino M., Durante M., Mueller-Mellin R., Reitz G., Rossi L., Shurshakov V., Sorbi M. (2007). Shielding from cosmic radiation for interplanetary missions: Active and passive methods. Radiat. Meas..

[148] Bamford R.A., Kellett B.J., Green J.L., Dong C., Airapetian V., Bingham R. (2022). How to create an artificial magnetosphere for Mars. Acta Astronaut..

[149] Binhi V.N., Prato F.S. (2017). Biological effects of the hypomagnetic field: An analytical review of experiments and theories. PLoS ONE.

[150] Hore P.J., Mouritsen H. (2016). The Radical-Pair Mechanism of Magnetoreception. Annu. Rev. Biophys..

[151] Blackman C.F., Benane S.G., House D.E. (1993). Evidence for direct effect of magnetic fields on neurite outgrowth. FASEB J..

[152] Xu J., Jarocha L.E., Zollitsch T., Konowalczyk M., Henbest K.B., Richert S., Golesworthy M.J., Schmidt J., Dejean V., Sowood D.J.C. (2021). Magnetic sensitivity of cryptochrome 4 from a migratory songbird. Nature.

[153] Binhi V.N. (2021). Random Effects in Magnetobiology and a Way to Summarize Them. Bioelectromagnetics.

[154] Hoff A.J., Rademaker H., Van Grondelle R., Duysens L.N.M. (1977). On the magnetic field dependence of the yield of the triplet state in reaction centers of photosynthetic bacteria. Biochim. Biophys. Acta BBA Bioenerg..

[155] Schulten K., Swenberg C.E., Weller A. (1978). A Biomagnetic Sensory Mechanism Based on Magnetic Field Modulated Coherent Electron Spin Motion. Z. Für Phys. Chem..

[156] Binhi V.N., Prato F.S. (2018). Rotations of macromolecules affect nonspecific biological responses to magnetic fields. Sci. Rep..

[157] Binhi V.N. (2019). Nonspecific magnetic biological effects: A model assuming the spin-orbit coupling. J. Chem. Phys..

[158] Kholodov Y.A. (1991). Bypassing the Sensory Organs?.

[159] Sarimov R., Alipov E.D., Belyaev I.Y. (2011). Fifty hertz magnetic fields individually affect chromatin conformation in human lymphocytes: Dependence on amplitude, temperature, and initial chromatin state. Bioelectromagnetics.

[160] Belyavskaya N.A. (2004). Biological effects due to weak magnetic field on plants. Adv. Space Res..

[161] Novitsky Y.I. (2016). The Effect of a Constant Magnetic Field on Plants.

[162] Breus T.K., Binhi V.N., Petrukovich A.A. (2016). Magnetic factor in solar-terrestrial relations and its impact on the human body: Physical problems and prospects for research. Phys. Uspekhi.

[163] Sarimov R.M., Serov D.A., Gudkov S.V. (2023). Biological effects of alternating magnetic fields. Bioelogy.

[164] Binhi V.N., Prato F.S. (2017). A physical mechanism of magnetoreception: Extension and analysis. Bioelectromagnetics.

[165] Binhi V.N. (1995). Nuclear spins in the primary mechanisms of biological action of magnetic fields. Biophysics.

[166] Buchachenko A.L. (2014). Magnetic field-dependent molecular and chemical processes in biochemistry, genetics and medicine. Russ. Chem. Rev..

[167] Binhi V.N. (2011). Principles of Electromagnetic Biophysics.

[168] Binhi V.N. (2002). Magnetobiology: Underlying Physical Problems.

[169] Yang X., Li Z., Polyakova T., Dejneka A., Zablotskii V., Zhang X. (2020). Effect of static magnetic field on DNA synthesis: The interplay between DNA chirality and magnetic field left-right asymmetry. FASEB Bioadv..

[170] Famiano M.A., Boyd R.N., Kajino T., Onaka T. (2018). Selection of amino acid chirality via neutrino interactions with 14N in crossed electric and magnetic fields. Astrobiology.

[171] Herd C.D.K., Blinova A., Simkus D.N., Huang Y., Tarozo R., Alexander C.M.O.D., Gyngard F., Nittler L.R., Cody G.D., Fogel M.L. (2011). Origin and Evolution of Prebiotic Organic Matter as Inferred from the Tagish Lake Meteorite. Science.

[172] Kvenvolden K., Lawless J., Pering K., Peterson E., Flores J., Ponnamperuma C., Kaplan I.R., Moore C. (1970). Evidence for Extraterrestrial Amino-acids and Hydrocarbons in the Murchison Meteorite. Nature.

[173] Binhi V.N., Chernavsi D.S. (2005). The stochastic resonance of magnetosomes fixed in the cytoskeleton. Biofizika.

[174] Kirschvink J.L., Winklhofer M., Walker M.M. (2010). Biophysics of magnetic orientation: Strengthening the interface between theory and experimental design. J. R. Soc. Interface.

[175] Milyaev V.A., Binhi V.N. (2006). On the physical nature of magnetobiological effects. Quantum Electron..

[176] Binhi V.N., Rubin A.B. (2007). Magnetobiology: The kT paradox and possible solutions. Electromagn. Biol. Med..

[177] Binhi V.N., Savin A.V. (2003). Effects of weak magnetic fields on biological systems: Physical aspects. Phys. Uspekhi.

[178] Binhi V.N. (1997). Mechanism of magnetosensitive binding of ions by certain proteins. Biophysics.

[179] Steiner U.E., Ulrich T. (2002). Magnetic field effects in chemical kinetics and related phenomena. Chem. Rev..

[180] Afanasyeva M.S., Taraban M.B., Purtov P.A., Leshina T.V., Grissom C.B. (2006). Magnetic Spin Effects in Enzymatic Reactions:  Radical Oxidation of NADH by Horseradish Peroxidase. J. Am. Chem. Soc..

[181] Shcherbakov I.A. (2022). Current Trends in the Studies of Aqueous Solutions. Phys. Wave Phenom..

[182] Lyakhov G.A., Man’ko V.I., Suyazov N.V., Shcherbakov I.A., Shermeneva M.A. (2022). Physical mechanisms of activation of radical reactions in aqueous solutions under mechanical and magnetic effect: Problem of singlet oxygen. Phys. Wave Phenom..

[183] Kattnig D.R., Solov’yov I.A., Hore P.J. (2016). Electron spin relaxation in cryptochrome-based magnetoreception. Phys. Chem. Chem. Phys..

[184] Gauger E.M., Rieper E., Morton J.J.L., Benjamin S.C., Vedral V. (2011). Sustained Quantum Coherence and Entanglement in the Avian Compass. Phys. Rev. Lett..

[185] Maeda K., Robinson A.J., Henbest K.B., Hogben H.J., Biskup T., Ahmad M., Schleicher E., Weber S., Timmel C.R., Hore P.J. (2012). Magnetically sensitive light-induced reactions in cryptochrome are consistent with its proposed role as a magnetoreceptor. Proc. Natl. Acad. Sci. USA.

[186] Binhi V.N. (2000). Amplitude and frequency dissociation spectra of ion-protein complexes rotating in magnetic fields. Bioelectromagnetics.

[187] Binhi V.N., Rubin A.B. (2022). Theoretical Concepts in Magnetobiology after 40 Years of Research. Cells.

[188] Lambert C.J. (2015). Basic concepts of quantum interference and electron transport in single-molecule electronics. Chem. Soc. Rev..

[189] Wan G., Hayden A.N., Iiams S.E., Merlin C. (2021). Cryptochrome 1 mediates light-dependent inclination magnetosensing in monarch butterflies. Nat. Commun..

[190] Gunkel M., Schoneberg J., Alkhaldi W., Irsen S., Noe F., Kaupp U.B., Al-Amoudi A. (2015). Higher-order architecture of rhodopsin in intact photoreceptors and its implication for phototransduction kinetics. Structure.

[191] Marshak D.W., Mills S.L. (2014). Short-wavelength cone-opponent retinal ganglion cells in mammals. Vis. Neurosci..

[192] Yang Z., Liu B., Su J., Liao J., Lin C., Oka Y. (2017). Cryptochromes Orchestrate Transcription Regulation of Diverse Blue Light Responses in Plants. Photochem. Photobiol..

[193] Gegear R.J., Foley L.E., Casselman A., Reppert S.M. (2010). Animal cryptochromes mediate magnetoreception by an unconventional photochemical mechanism. Nature.

[194] Iwaniuk A., Heyers D., Manns M., Luksch H., Güntürkün O., Mouritsen H. (2007). A Visual Pathway Links Brain Structures Active during Magnetic Compass Orientation in Migratory Birds. PLoS ONE.

[195] Ozturk N. (2017). Phylogenetic and Functional Classification of the Photolyase/Cryptochrome Family. Photochem. Photobiol..

[196] Pooam M., Arthaut L.-D., Burdick D., Link J., Martino C.F., Ahmad M. (2018). Magnetic sensitivity mediated by the Arabidopsis blue-light receptor cryptochrome occurs during flavin reoxidation in the dark. Planta.

[197] Hammad M., Albaqami M., Pooam M., Kernevez E., Witczak J., Ritz T., Martino C., Ahmad M. (2020). Cryptochrome mediated magnetic sensitivity in Arabidopsis occurs independently of light-induced electron transfer to the flavin. Photochem. Photobiol. Sci..

[198] Ahmad M., Cashmore A.R. (1993). HY4 gene of A. thaliana encodes a protein with characteristics of a blue-light photoreceptor. Nature.

[199] Chaves I., Pokorny R., Byrdin M., Hoang N., Ritz T., Brettel K., Essen L.-O., van der Horst G.T.J., Batschauer A., Ahmad M. (2011). The Cryptochromes: Blue Light Photoreceptors in Plants and Animals. Annu. Rev. Plant Biol..

[200] Deviers J., Cailliez F., de la Lande A., Kattnig D.R. (2022). Anisotropic magnetic field effects in the re-oxidation of cryptochrome in the presence of scavenger radicals. J. Chem. Phys..

[201] Thoradit T., Thongyoo K., Kamoltheptawin K., Tunprasert L., El-Esawi M.A., Aguida B., Jourdan N., Buddhachat K., Pooam M. (2023). Cryptochrome and quantum biology: Unraveling the mysteries of plant magnetoreception. Front. Plant Sci..

[202] Richards O.W., Davies R.G. (1977). Imms’ General Textbook of Entomology: Structure, Physiology and Development.

[203] Binhi V.N. (2018). A limit in the dynamic increase in the accuracy of group migration. BioSystems.

[204] Xu P., Xiang Y., Zhu H., Xu H., Zhang Z., Zhang C., Zhang L., Ma Z. (2009). Wheat Cryptochromes: Subcellular Localization and Involvement in Photomorphogenesis and Osmotic Stress Responses. Plant Physiol..

[205] Stenkamp D.L. (2015). Development of the Vertebrate Eye and Retina. Prog. Mol. Biol. Transl. Sci..

[206] Cochran W.W., Mouritsen H., Wikelski M. (2004). Migrating Songbirds Recalibrate Their Magnetic Compass Daily from Twilight Cues. Science.

[207] Moore F.R. (1985). Integration of environmental stimuli in the migratory orientation of the savannah sparrow (Passerculus sandwichensis). Anim. Behav..

[208] Güler A.D., Ecker J.L., Lall G.S., Haq S., Altimus C.M., Liao H.-W., Barnard A.R., Cahill H., Badea T.C., Zhao H. (2008). Melanopsin cells are the principal conduits for rod–cone input to non-image-forming vision. Nature.

[209] Solov’yov I.A., Mouritsen H., Schulten K. (2010). Acuity of a cryptochrome and vision-based magnetoreception system in birds. Biophys. J..

[210] Müller P., Yamamoto J., Martin R., Iwai S., Brettel K. (2015). Discovery and functional analysis of a 4th electron-transferring tryptophan conserved exclusively in animal cryptochromes and (6-4) photolyases. Chem. Commun..

[211] Cailliez F., Müller P., Firmino T., Pernot P., de la Lande A. (2016). Energetics of Photoinduced Charge Migration within the Tryptophan Tetrad of an Animal (6–4) Photolyase. J. Am. Chem. Soc..

[212] Romero E., Augulis R., Novoderezhkin V.I., Ferretti M., Thieme J., Zigmantas D., van Grondelle R. (2014). Quantum coherence in photosynthesis for efficient solar-energy conversion. Nat. Phys..

[213] Binhi V. (2008). Do naturally occurring magnetic nanoparticles in the human body mediate increased risk of childhood leukaemia with EMF exposure?. Int. J. Radiat. Biol..

[214] Blakemore R. (1975). Magnetotactic bacteria. Science.

[215] Kirschvink J.L., Kobayashi-Kirschvink A., Woodford B.J. (1992). Magnetite biomineralization in the human brain. Proc. Natl. Acad. Sci. USA.

[216] Bazylinski D.A., Lefèvre C.T., Schüler D., Rosenberg E., DeLong E.F., Lory S., Stackebrandt E., Thompson F. (2013). Magnetotactic Bacteria. The Prokaryotes: Prokaryotic Physiology and Biochemistry.

[217] Bazylinski D.A., Schübbe S. (2007). Controlled biomineralization by and applications of magnetotactic bacteria. Adv. Appl. Microbiol..

[218] Zeytuni N., Ozyamak E., Ben-Harush K., Davidov G., Levin M., Gat Y., Moyal T., Brik A., Komeili A., Zarivach R. (2011). Self-recognition mechanism of MamA, a magnetosome-associated TPR-containing protein, promotes complex assembly. Proc. Natl. Acad. Sci. USA.

[219] Wang X., Li Y., Zhao J., Yao H., Chu S., Song Z., He Z., Zhang W. (2020). Magnetotactic bacteria: Characteristics and environmental applications. Front. Environ. Sci. Eng..

[220] Riese C.N., Wittchen M., Jérôme V., Freitag R., Busche T., Kalinowski J., Schüler D. (2022). The transcriptomic landscape of Magnetospirillum gryphiswaldense during magnetosome biomineralization. BMC Genom..

[221] Grassi-Schultheiss P.P., Heller F., Dobson J. (1997). Analysis of magnetic material in the human heart, spleen and liver. BioMetals.

[222] Schultheiss-Grassi P.P., Dobson J. (1999). Magnetic analysis of human brain tissue. Biometals.

[223] Mikhaylova A., Davidson M., Toastmann H., Channell J.E.T., Guyodo Y., Batich C., Dobson J. (2005). Detection, identification and mapping of iron anomalies in brain tissue using X-ray absorption spectroscopy. J. R. Soc. Interface.

[224] Stovbun S.V., Zlenko D.V., Bukhvostov A.A., Vedenkin A.S., Skoblin A.A., Kuznetsov D.A., Buchachenko A.L. (2023). Magnetic field and nuclear spin influence on the DNA synthesis rate. Sci. Rep..

[225] Binhi V.N. (2005). Stochastic dynamics of magnetic nanoparticles and a mechanism of biological orientation in the geomagnetic field. Physics.

[226] Walker M.M., Dennis T.E., Kirschvink J.L. (2002). The magnetic sense and its use in long-distance navigation by animals. Curr. Opin. Neurobiol..

[227] Wiltschko W., Munro U., Wiltschko R., Kirschvink J.L. (2002). Magnetite-based magnetoreception in birds: The effect of a biasing field and a pulse on migratory behavior. J. Exp. Biol..

[228] Davila A.F., Fleissner G., Winklhofer M., Petersen N. (2003). A new model for a magnetoreceptor in homing pigeons based on interacting clusters of superparamagnetic magnetite. Phys. Chem. Earth.

[229] Hanzlik M., Heunemann C., Holtkamp-Rotzler E., Winklhofer M., Petersen N., Fleissner G. (2000). Superparamagnetic magnetite in the upper beak tissue of homing pigeons. BioMetals.

[230] Qin S., Yin H., Yang C., Dou Y., Liu Z., Zhang P., Yu H., Huang Y., Feng J., Hao J. (2015). A magnetic protein biocompass. Nat. Mater..

[231] Mandilaras K., Missirlis F. (2012). Genes for iron metabolism influence circadian rhythms in Drosophila melanogaster. Metallomics.

[232] Meister M. (2016). Physical limits to magnetogenetics. eLife.

[233] Dunlop D.J. (1972). Magnetite: Behavior near the Single-Domain Threshold. Science.

[234] Ramsay J.L., Kattnig D.R. (2023). Magnetoreception in cryptochrome enabled by one-dimensional radical motion. AVS Quantum Sci..

[235] McCraty R., Zayas M.A. (2014). Cardiac coherence, self-regulation, autonomic stability, and psychosocial well-being. Front. Psychol..

[236] Safronova V.G., Vulfius C.A., Astashev M.E., Tikhonova I.V., Serov D.A., Jirova E.A., Pershina E.V., Senko D.A., Zhmak M.N., Kasheverov I.E. (2021). α9α10 nicotinic acetylcholine receptors regulate murine bone marrow granulocyte functions. Immunobiology.

[237] Siniavin A., Streltsova M., Kudryavtsev D., Shelukhina I., Utkin Y., Tsetlin V. (2020). Activation of α7 Nicotinic Acetylcholine Receptor Upregulates HLA-DR and Macrophage Receptors: Potential Role in Adaptive Immunity and in Preventing Immunosuppression. Biomolecules.

[238] Barberà M., Collantes-Alegre J.M., Martínez-Torres D. (2021). Mapping and quantification of cryptochrome expression in the brain of the pea aphid Acyrthosiphon pisum. Insect Mol. Biol..

[239] Vechtomova Y., Telegina T., Buglak A., Kritsky M. (2021). UV Radiation in DNA Damage and Repair Involving DNA-Photolyases and Cryptochromes. Biomedicines.

